# Neuroprotective effect of early remote ischaemic post-conditioning after out-of-hospital cardiac arrest (RIPOST-CA): study protocol for a randomised, single-blinded, monocentric, controlled trial

**DOI:** 10.1016/j.resplu.2026.101503

**Published:** 2026-09-24

**Authors:** Jonathan Chelly, Romain Cheyssac, Thibaut Bertrand, Vincent Gardan, Asmaa Jobic, Masoud Rahmati, Romain Lambert, Julie Durif, Vincent Sapin, Alain Cariou, Armel Dell Aria, Laurent Ducros

**Affiliations:** a Réanimation Polyvalente, Centre Hospitalier Intercommunal de Toulon La Seyne-sur-Mer, Toulon, France; b Centre d’Étude et de Recherche sur les Systèmes de Soins et la qualité de vie (CEReSS), Aix Marseille Université, Marseille, France; c Délégation à la Recherche Clinique et à l’Innovation du Groupement Hospitalier de Territoire du Var, Centre Hospitalier Intercommunal de Toulon La Seyne-sur-Mer, Toulon, France; d AfterROSC Research Network, France; e SAMU 83, Centre Hospitalier Intercommunal de Toulon La Seyne-sur-Mer, Toulon, France; f Plateforme BIOPROPLEX, Biochimie et Génétique Moléculaire, Centre Hospitalier Universitaire de Clermont Ferrand, Clermont Ferrand, France; g Médecine Intensive Réanimation, Hôpital Cochin, AP-HP Centre Université Paris Cité, Paris, France; h Service de Biochimie, Centre Hospitalier Intercommunal de Toulon La Seyne-sur-Mer, Toulon, France

**Keywords:** Out-of-hospital cardiac arrest, Remote ischaemic conditioning, Hypoxic-ischaemic brain injury, Neurofilament light chain, Neuroprotection, Ischaemia–reperfusion injury

## Abstract

**Introduction:**

Hypoxic-ischaemic brain injury is the leading cause of death and long-term disability after out-of-hospital cardiac arrest and is driven by the ischaemia–reperfusion syndrome, for which no specific treatment exists. Remote ischaemic post-conditioning (brief, repeated cycles of limb ischaemia and reperfusion delivered with a pneumatic cuff), is protective across ischemia–reperfusion conditions. We aim to assess whether early conditioning reduces hypoxic-ischaemic brain injury after out-of-hospital cardiac arrest.

**Methods:**

This single-centre, single-blinded, randomised (1:1) controlled trial with blinded assessment of the outcome, enrols 48 comatose adults resuscitated from out-of-hospital cardiac arrest. Participants receive standard post-resuscitation care with three sham cuff sessions, or standard care plus three sessions of conditioning (thigh cuff, four cycles of 5-min inflation at 200 mmHg alternating with 5-min deflation), the first within 4 h of return of spontaneous circulation and repeated at the 12th and 24th hour. Randomisation is stratified by initial rhythm (shockable versus non-shockable). The primary endpoint is the change in serum neurofilament light chain between the 6th and 72nd hour after return of spontaneous circulation. Secondary endpoints include daily Sequential Organ Failure Assessment scores (days 1–3), serum neuron-specific enolase and protein S100-B kinetics, in-intensive care unit mortality by cause, and a favourable neurological outcome (modified Rankin Scale 0–3) at intensive care unit discharge and at 90 days.

**Conclusion:**

RIPOST-CA is the first randomised trial to evaluate early remote ischemic post-conditioning on brain-injury biomarkers after out-of-hospital cardiac arrest. Its results could inform larger clinical trials of this simple, non-invasive intervention.

**Trial registration number:** NCT06473207.

## Introduction

Out-of-hospital cardiac arrest (OHCA) remains a major public-health problem, accounting for an estimated 275,000 and 350,000 sudden deaths each year in Europe and the United States, respectively.^1^, ^2^ OHCA patients’ outcome remains poor and has improved only modestly over recent decades, with survival-to-hospital-discharge ranging from around 2% for cardiac arrests presenting with asystole to about 30% for those with an initial shockable rhythm.^3^, ^4^ Only a minority of patients admitted alive to intensive care unit (ICU) after a resuscitated OHCA will ultimately survive with limited or no neurological sequelae. Approximately two-thirds of in-ICU deaths after an OHCA are attributable to brain injury.^5^ This anoxic-ischaemic injury is a central component of the post-cardiac arrest syndrome and is driven principally by the ischaemia–reperfusion syndrome.^6^ Return of spontaneous circulation (ROSC) triggers a systemic inflammatory response and the release of cytotoxic mediators throughout organs, including the brain.^6^, ^7^ Beyond the supportive measures recommended by guidelines (haemodynamic optimisation, maintenance of cerebral homeostasis and prevention of secondary systemic brain insults), no specific treatment has been shown to reduce ischaemia–reperfusion and brain injuries after cardiac arrest.^7^ Reducing ischaemia–reperfusion injury therefore remains a major therapeutic target,^8^ and a recent systematic review and meta-analysis confirmed that no pharmacological strategy has convincingly improved functional outcome in comatose survivors of cardiac arrest.^9^

Remote ischaemic conditioning induces brief, repeated cycles of limb ischaemia and reperfusion with a pneumatic cuff. When applied after the insult, it is termed remote ischaemic *post*-conditioning. Although its mechanisms are not fully understood, preclinical work has identified humoral, neural and anti-inflammatory pathways that confer cytoprotection across organs, including the brain, from this simple, non-invasive and inexpensive intervention.^10^, ^11^ Inhibition of opening of the mitochondrial permeability transition pore (a key effector of ischaemia–reperfusion cell death) is one of the protective mechanisms identified when conditioning is delivered early after the insult.^10^, ^12^ The efficacy of remote ischaemic post-conditioning also appears to depend on the “dose” delivered, including the number and duration of cycles.^13^ In humans, remote ischaemic post-conditioning has yielded promising results across several conditions: myocardial infarction and cardiac surgery,^14^, ^15^, ^16^ acute ischaemic stroke,^17^, ^18^, ^19^, ^20^ severe traumatic brain injury,^21^ and more recently in septic shock with promising results without notable adverse events.^22^, ^23^ Regarding cardiac arrest, preclinical studies have shown that remote ischaemic conditioning applied during or immediately after cardiopulmonary resuscitation reduces ischaemic brain lesions and improves myocardial recovery and neurological prognosis.^24^, ^25^ A first clinical study recently demonstrated the feasibility and safety of remote ischaemic post-conditioning initiated as early as the prehospital phase in resuscitated OHCA.^26^ Remote ischaemic post-conditioning has therefore been proposed as a realistic candidate intervention in emergency and critical-care medicine.^27^, ^28^

The severity of anoxic-ischaemic brain injury can be quantified with validated, brain-specific blood biomarkers reflecting anatomically distinct lesions: neurofilament light chain (NfL), neuron-specific enolase (NSE), and protein S100-B, for axonal, neuronal, and astrocytic injury, respectively.^29^, ^30^ Our primary objective is to assess the effect of early remote ischaemic post-conditioning, compared with a sham procedure, on the reduction of ischaemia–reperfusion axonal brain injury in patients resuscitated from OHCA. We hypothesise that early remote ischaemic post-conditioning can reduce anoxic-ischaemic brain injury and secondarily reduce organ failure and improve neurological outcome.

## Methods

### Study setting

The Remote Ischemic POST-conditioning on Cardiac Arrest brain injury (RIPOST-CA) trial is a prospective, single-centre, single-blinded, randomised (1:1) controlled superiority trial, with blinded assessment of the outcome, comparing two arms. This trial is supported by a research grant from the French Intensive Care Society and by internal funding from the *Centre Hospitalier Intercommunal Toulon La Seyne-sur-Mer*, a non-university tertiary care centre. The funder (French Intensive Care Society) had or will have any role in the study design, conduct, data collection, analysis, interpretation, or reporting, or in the decision to submit the manuscript for publication. The initial study protocol was approved by a French Ethics Committee on 13 March 2024 (*Comité de Protection des Personnes Est IV*, Strasbourg, France) and will be performed in accordance with the Declaration of Helsinki. It was prospectively registered at ClinicalTrials.gov on 17 June 2024 (NCT06473207). An amendment approved by the same Ethics Committee on 4 May 2026 modified the biomarker assessment window (48th or 72nd hour, according to outcome), refined a non-inclusion criterion and extended enrolment to 36 months. The full protocol is provided in supplementary material. This trial follows the Standard Protocol Items: Recommendations for Interventional Trials (SPIRIT) guidance (see the SPIRIT checklist in the supplementary material).^31^

### Patients and public involvement

Patients or the public were not involved in the design, conduct, reporting or dissemination of this study.

### Population and study protocol

Eligible patients (see the flow chart provided in Fig. 1) are comatose (Glasgow Coma Scale <8) adults admitted to a 24-bed ICU of a French tertiary centre after an OHCA of any initial rhythm, who meet all inclusion and no exclusion criteria (Table 1). To maximise any protective effect, the allocated treatment is applied as early as possible after eligibility is confirmed (if possible, during the prehospital phase, otherwise on ICU admission), and always within 4 h after ROSC.

**Fig. 1:**
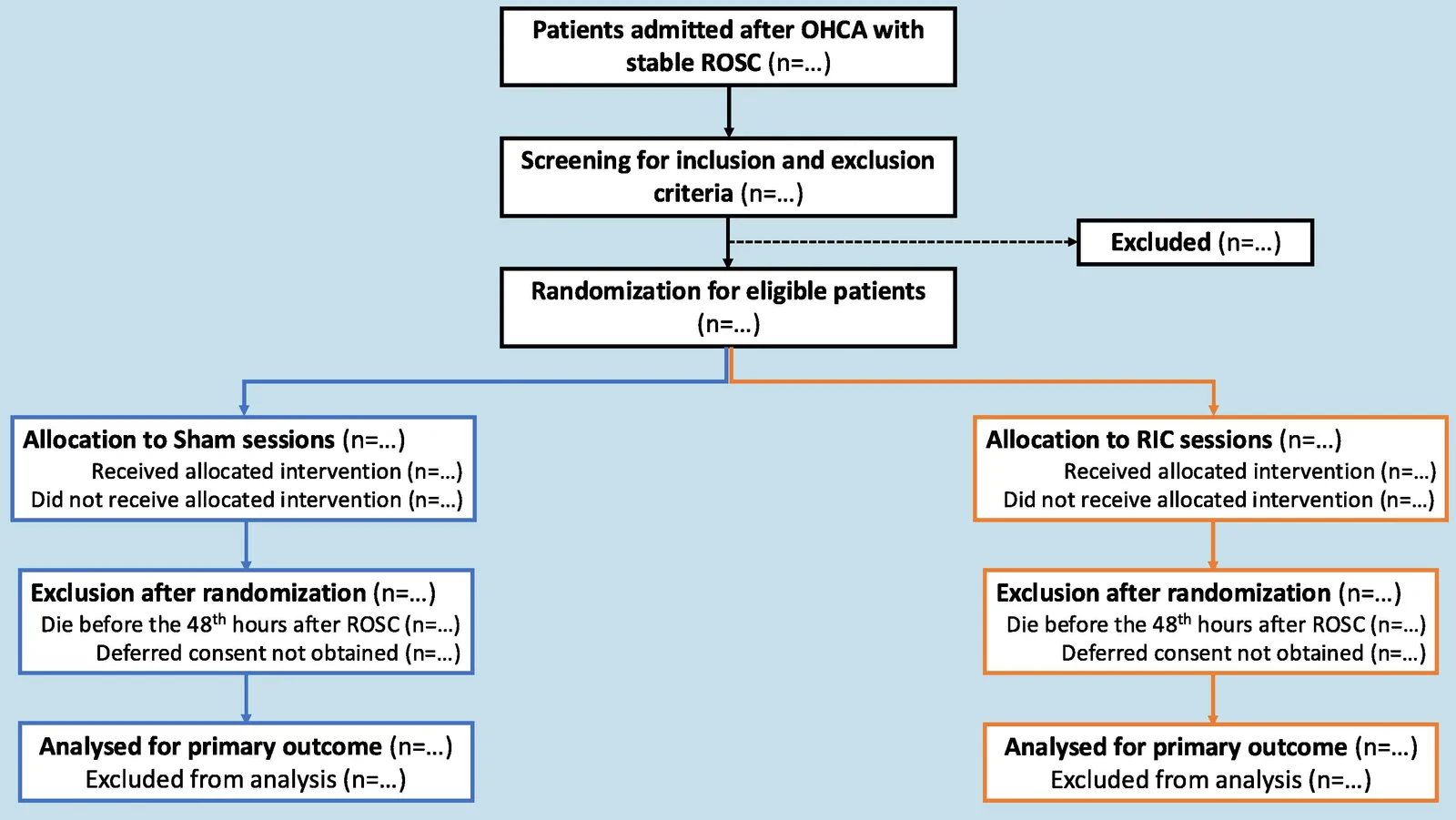
**Participant flow diagram according to the Consolidated Standards of Reporting Trials statement 2025**. OHCA: out-of-hospital cardiac arrest; ROSC: return of spontaneous circulation; RIC: remote ischaemic post-conditioning.

**Table 1:** Inclusion and non-inclusion criteria of the RIPOST-CA trial.

**Inclusion criteria**	**Non-inclusion criteria**
A comatose (GCS <8) adult admitted to ICU for an OHCA (shockable or non-shockable) with stable ROSC (≥20 min) *and* Availability of a lower limb free of any vascular line or blood-pressure cuff *and* Randomisation and application of the first remote ischemic conditioning (or sham) session within 4 hrs after ROSC *and* Consent from a relative, or inclusion under the emergency procedure	Age <18 years, or pregnancy *or* Poor baseline functional status (mRS 4–5) *or* Estimated no flow + low flow > 60 min *or* Unwitnessed cardiac arrest with initial asystole *or* Refractory arrest (no ROSC considered stable) *or* Cardiac arrest of traumatic, haemorrhagic, primary acute cerebral (stroke, head trauma), or hanging origin *or* Persistent MAP <65 mmHg despite adequate fluid resuscitation and vasopressor/inotropic support *or* Uncontrolled active haemorrhage *or* Contraindication or inability to place a pneumatic cuff on either lower limb (amputation, vascular line on both limbs, cuff size incompatible with severe obesity) *or* Veno-arterial ECMO for refractory cardiac arrest or cardiogenic shock before inclusion *or* patient already included in this study *or* inclusion in another interventional study which purpose is to improve prognosis after cardiac arrest *or* Adults under a legal protection measure (safeguard of justice, guardianship or curatorship), or without French social security

ECMO: extracorporeal membrane of oxygenation; GCS: Glasgow coma scale; ICU: intensive care unit; Low flow: interval between basic life support and ROSC; MAP: mean arterial pressure; mRS: modified Rankin scale; No flow: interval between CA occurrence and basic life support; OHCA: out-of-hospital cardiac arrest; ROSC: return of spontaneous circulation.

Patients are allocated to one of two arms (Fig. 2). In the intervention arm (Conditioning group), one remote ischemic post-conditioning session consists of inflating a thigh cuff to 200 mmHg for 5 min then deflating it for 5 min, repeated four times consecutively (Fig. 3). The first session starts within the first hour after inclusion and the next two at the 12th and 24th hour after ROSC. In the control arm (Sham group), the cuff is applied at the same sites and time-points but is left uninflated for 40 min, preserving blinding of relatives. In both arms, a session is postponed if arterial hypotension is present until haemodynamic stabilisation. For both arms, post-resuscitation care follows current European Resuscitation Council and European Society of Intensive Care Medicine guidelines^7^ including haemodynamic optimisation, sedation management, controlled oxygenation and ventilation, and targeted temperature management. The prediction of neurological outcome and any decision to withdraw life-sustaining therapy follow routine practice at the centre in accordance with current guidelines.^7^

**Fig. 2:**
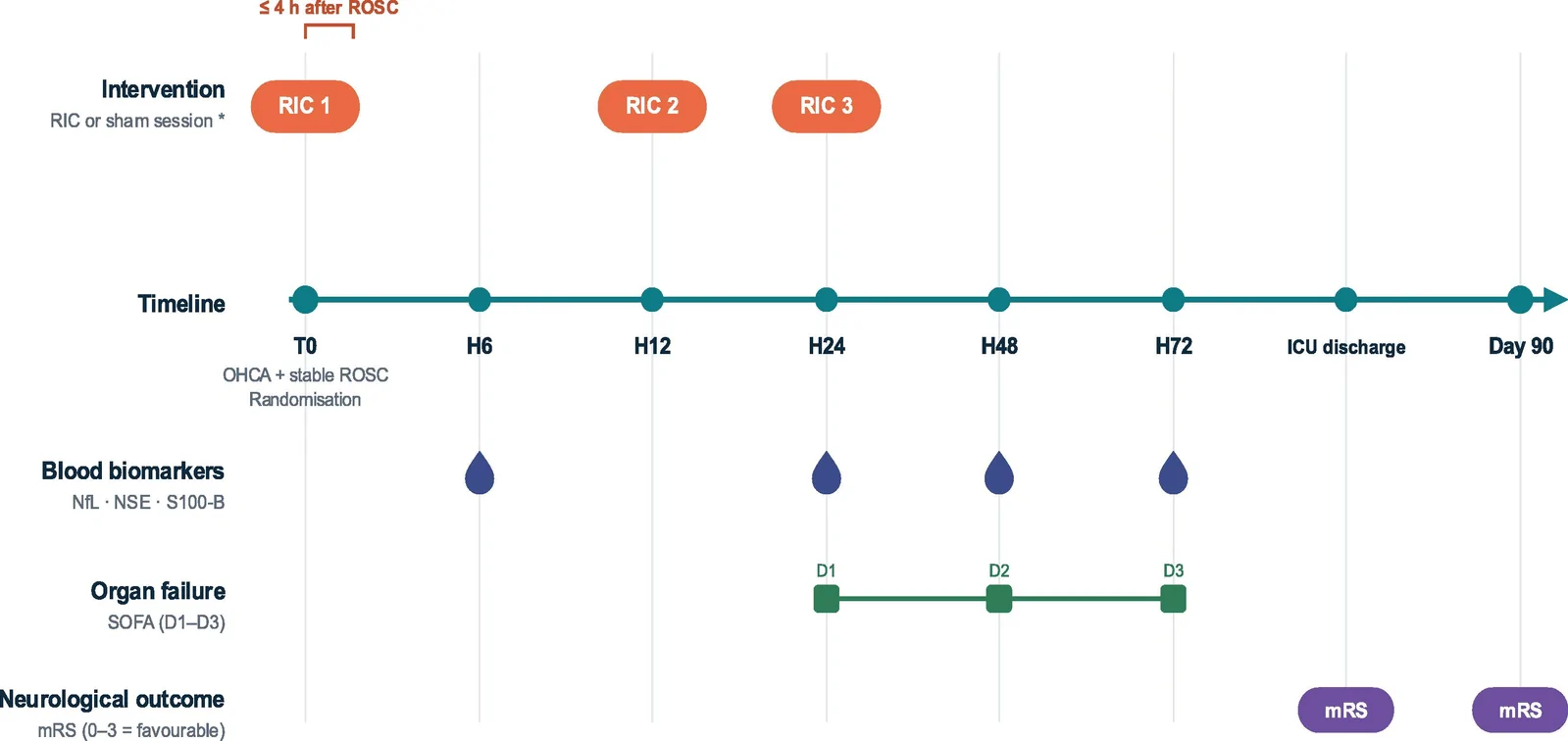
**Data collection plan and follow up according to SPIRIT statement. T0 from T72 are defined as the timepoints (in hours) starting from the first stable ROSC (T0) to 72 h (T72)**. ICU: intensive care unit; RIC: remote ischaemic post-conditioning; NfL: neurofilament light chain; NSE: neuron-specific enolase; SOFA: sepsis-organ related failure assessment score; mRS: modified Rankin scale; ROSC: return of spontaneous circulation; OHCA: out-of-hospital cardiac arrest. *Intervention arm: three remote ischaemic conditioning sessions. Control arm: identical sham session (uninflated cuff) at the same time-points.

**Fig. 3:**
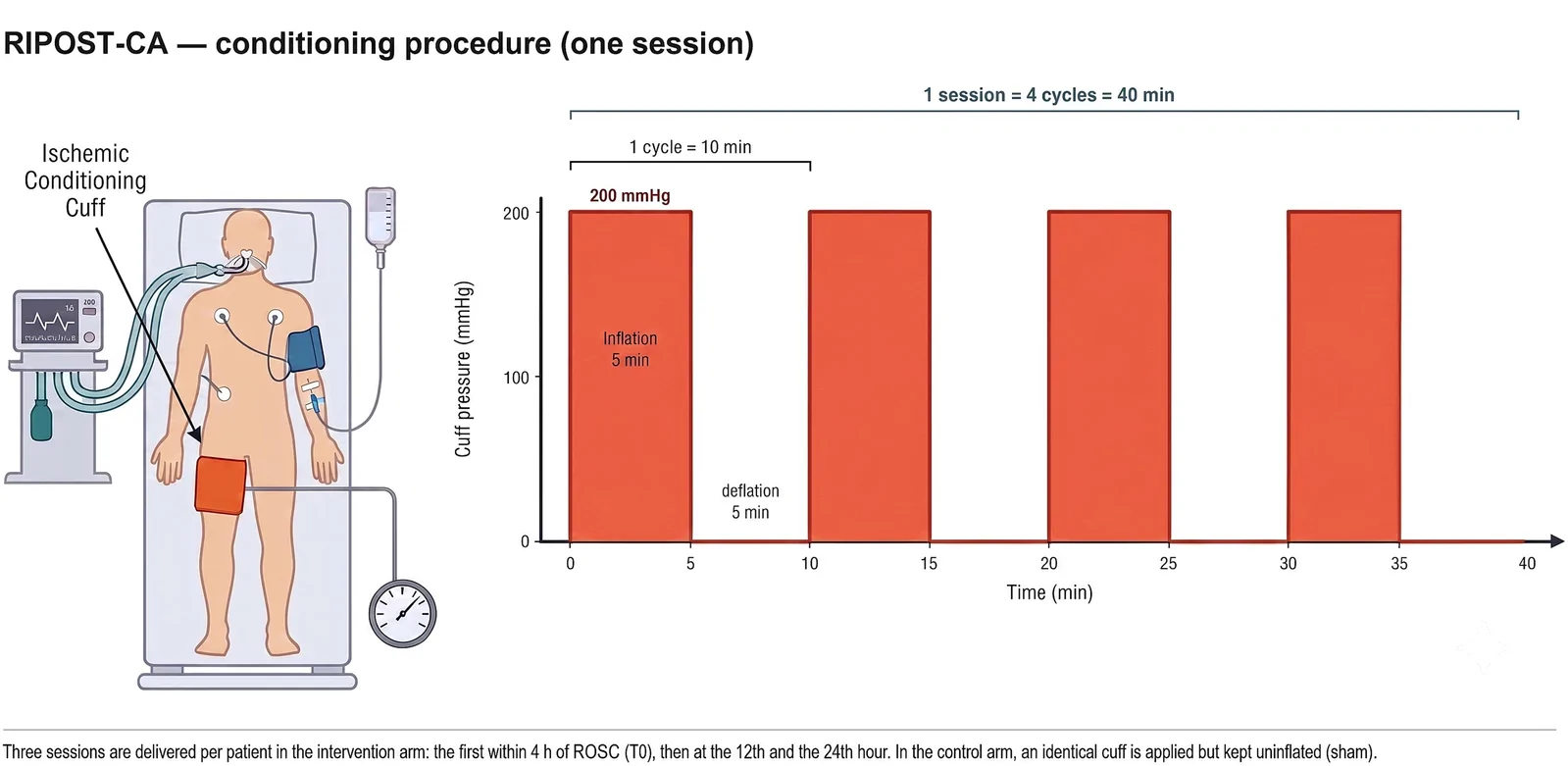
**Remote ischemic conditioning procedure. Three sessions are delivered per patient in the intervention arm: the first within 4 h after return of spontaneous circulation, then at the 12th and the 24th hour. In the control arm, an identical cuff is applied but kept uninflated (sham)**.

Randomisation is centralised and computer-based, with allocation concealment. The allocation sequence (1:1) was generated in advance by the trial statistician using permuted blocks of random sizes stratified by the initial rhythm (shockable versus non-shockable) and is embedded in the secure electronic case report form. The sequence is concealed from the investigators: at inclusion, once eligibility is confirmed and the patient enrolled, the system discloses the allocation for that patient only. Because the intervention requires the bedside team to inflate or not the cuff, the trial is open-label with respect to the treating team. Patients and relatives are blinded throughout the study. Outcome assessment is also blinded: laboratory personnel performing the biomarker assays and the modified Rankin scale (mRS) assessor are unaware of allocation. Investigators and the treating team remain blinded to all study biomarker results, except NSE at the 24th, 48th and 72nd hour, which is reported as part of the guideline-recommended multimodal neuroprognostication.^7^

### Data collection and outcome variables

For each participant, data are pseudonymised and collected in a centralised electronic case report form according to Utstein recommendations.^32^ Prehospital and in-hospital variables include patient characteristics (age, sex, body mass index, comorbidities, pre-arrest functional status estimated with the mRS, and the Clinical Frailty Scale), arrest circumstances, no-flow and low-flow intervals, initial rhythm, prehospital management, presumed cause of arrest, and daily clinical and biological parameters up to the 72nd hour. Vital status and the mRS are recorded at ICU discharge and at 90 days, collected during the hospital stay or by telephone interview with the patient or a relative, if appropriate.

Blood samples for NfL, NSE, and S100B are drawn at the 6th, 24th, 48th and 72nd hour after ROSC. NfL concentrations will be quantified in all participants using the Simple Plex® cartridge-based immunoassay on the Ella™ platform (ProteinSimple, San Jose, CA, USA), S100B concentrations using the Elecsys® S100B electrochemiluminescence immunoassay (Roche Diagnostics, Mannheim, Germany) on a Cobas® e801 platform, and NSE concentrations using the Elecsys® electrochemiluminescence immunoassay (Roche Diagnostics, Meylan, France) on a Cobas® pro platform. A laboratory physician will systematically assess the haemolysis index before reporting NSE values to ensure accurate NSE levels.

The primary endpoint is the change in NfL between the 6th and the 72nd hour after ROSC. Patients who die before the 48th hour are excluded from the primary analysis. For patients who die or are discharge from the hospital between the 48th and 72nd hour, NfL values are considered up to the 48th hour. Secondary endpoints, compared between arms, are as follows: (1) the total daily Sequential Organ Failure Assessment score (SOFA) from day 1 to day 3 and the per-organ SOFA over the same period (patients who die are assigned the maximum score for total SOFA and each organ sub-score); (2) the proportion of patients with a peak NfL >500 pg/mL; (3) the change in serum NSE between the 6th and the 72nd hour after ROSC (or the 48th hour in case of early death or discharged from the hospital), and the proportion of patients with a peak NSE > 60 µg/L; (4) the change in serum protein S100B between the 6th and the 72nd hour after ROSC (or the 48th hour in case of early death or discharge from the hospital); (5) the rate of in-ICU death attributed to multiple organ failure and the rate of in-ICU death attributed to neurological failure; (6) the proportion of patients with a favourable neurological outcome (defined as a mRS of 0–3), at ICU discharge and at 90 days after OHCA; and (7) the proportion of post-cardiac arrest event occurrence within the 24 h following cardiac arrest (including unexpected cardiac arrest in the ICU, iatrogenic events, limb ischemia, occlusive syndrome, digestive ischemia, and thromboembolic complications).

### Statistical method

Missing values will be reported without imputation, except for the pre-specified worst-case rule assigning the maximum SOFA to patients who die. The full analysis set comprises all randomised patients with valid informed consent, described and compared by arm (intention-to-treat). The modified intention-to-treat population comprises randomised patients surviving to the 48th hour with at least one NfL value between the 6th and the 72nd (or 48th) hour, analysed as randomised. It serves for a sensitivity analysis of the primary endpoint. The per-protocol population comprises patients who received the allocated procedures without major deviation and evaluable for the primary endpoint. It is used for the primary and secondary analysis, consistent with estimating the biological efficacy of remote ischemic post-conditioning as delivered. Statistical analysis will be performed on fully anonymised data.

For the primary endpoint, the 6-to-72-h NfL change (or the 48th hour for patients dying in that interval) and the peak NfL are compared between arms (per-protocol) using Wilcoxon–Mann–Whitney tests, with the Hodges-Lehmann estimate of the median difference and its 95% confidence interval (CI) as effect size. The modified intention-to-treat population serves as sensitivity analysis. Associations between NfL kinetics and other biomarkers (NSE, S100B) or SOFA are explored with Spearman correlation coefficients. NfL changes across sampling times and between arms are analysed with a linear mixed-effects model, with fixed effect for arm, time, and the arm-by-time interaction, a random intercept for patient, and adjustment for the randomization stratification factor. NfL values are log-transformed before modelling to satisfy normality assumptions, checked with the Shapiro-Wilk test and QQ-plots on model residuals. Applied to the per-protocol population (modified intention-to-treat as sensitivity analysis), it accommodates incomplete follow-up without imputation. The arm-by-time interaction is the primary test of a time-varying treatment effect.

Secondary endpoints (per-protocol): SOFA and NSE/S100B kinetics are compared with Wilcoxon–Mann–Whitney tests or linear mixed models; proportions (peak NfL >500 pg/mL, peak NSE >60 µg/L), death rates by cause, favourable outcome (mRS 0–3) at ICU discharge and day 90, and post-cardiac arrest event with chi-square (or Fisher’s exact) tests, with odds ratios (95% confidence interval) from logistic regression adjusted for the stratification factor, with the Wald test used for the significance of model coefficient. All tests are two-sided, with a significance level of 0.05. Given their exploratory nature, secondary endpoints are not adjusted for multiplicity and are interpreted as hypothesis-generating.

The sample size was determined based on the primary endpoint, to detect a 50% reduction in NfL change in the intervention arm. Based on Moseby-Knappe et al.^33^ and Adler et al.,^34^ 72-h NfL was assumed to be 3250 pg/mL (±2000) (control) and 1625 pg/mL (±1000) (intervention). With a two-sided alpha of 5%, 16 patients per arm provide at least 80% power. Stratified randomization should balance 6-h NfL between arms, so the 6-to-72-h change is comparable to these 72-h values. Assuming up to 50% of patients die before the 48th hour and are non-evaluable, 24 patients per arm (48 total) are needed over a 36-month enrolment period.

### Safety, adverse events and data monitoring

The theoretical risks of remote ischemic post-conditioning are rare: transient aggravation of pre-existing limb ischaemia, transient hypotension (reversible on deflation), or occlusion of a vascular line placed on the conditioned limb. They are minimised by the non-inclusion criteria (Table 1) and by postponing any session during hypotension. Previous remote ischemic post-conditioning studies across critical conditions, including the first feasibility study after OHCA, reported excellent tolerance without notable adverse effects when contraindications were respected.^22^, ^26^ Adverse events are recorded and serious adverse events managed and reported per French regulations. No interim analysis is planned, and a single final analysis will be performed once all patients will complete participation. Given the procedure’s tolerance and the absence of interim analysis, no data safety monitoring board was constituted, a decision approved by the Ethics Committee.

### Ethics, dissemination, and data availability

Because treatment must start within 4 h of ROSC, the Ethics Committee approved an emergency inclusion procedure with deferred informed consent of the patient and next of kin, in accordance with French legislation: inclusion follows a relative’s consent or, if no relative is reachable, the investigating physician’s emergency decision. As soon as possible, relatives and then subsequently the patient (upon regaining capacity) are informed and written consent for continued participation is sought. In case of consent withdrawal, data collected up to that point are retained for analysis in accordance with applicable regulations. Results will be disseminated through conferences and peer-reviewed publications, following the Consolidated Standards of Reporting Trials statement^35^ (CONSORT checklist is provided in the supplementary material). A fully anonymised dataset generated during this study will be available from the corresponding author upon reasonable request.

## Discussion

RIPOST-CA is, to our knowledge, the first randomised controlled trial to assess the effect of early remote ischemic post-conditioning, compared with standard care, on cerebral biomarkers in patients resuscitated from OHCA.

Few clinical studies have evaluated remote ischemic post-conditioning after resuscitated OHCA. A single-centre feasibility study demonstrated its feasibility and safety in the emergency department.^26^ A South Korean single-centre study (NCT03093948), applying remote ischemic post-conditioning to both thighs at the start of targeted temperature management, completed enrolment of 58 OHCA patients but remains unpublished to date. Importantly, a second, larger French trial is currently ongoing: RECO-OHCA (NCT06306625), a multicentre study recruiting 220 patients since June 2024. RECO-OHCA and RIPOST-CA differ substantially and are complementary rather than being redundant: RECO-OHCA compares brachial (upper-limb) remote ischemic post-conditioning with sham in patients aged 18–80, with a clinical composite primary endpoint at 72 h after admission (all-cause death, multiple organ failure, or severe neurological failure) and secondary endpoints including serial SOFA, serum NSE at 48–72 h, and the Cerebral Performance Category at day 90. Together, the two trials address the same gap from complementary angles, and their combined results could strengthen the rationale for larger multicentre trials of remote ischemic post-conditioning in cardiac arrest.

### Limitations

Several limitations should be highlighted. First, RIPOST-CA is an exploratory single-centre trial, which may limit external validity. In the absence of published preclinical or clinical data on remote ischemic post-conditioning dosing and effect size in cardiac arrest, no prior study could ground the primary hypothesis. The assumed 50% NfL reduction is thus ambitious and empirical, and the sample size is that of a pilot trial intended to inform rather than definitively establish remote ischemic post-conditioning efficacy, possibly underpowered for more modest effects. Second, the primary endpoint is a surrogate biomarker rather than a patient-centred functional outcome, and clinical outcomes are only secondary. This choice is nonetheless supported by NfL’s high sensitivity and specificity for post-cardiac arrest brain injury severity and by kinetics that discriminate outcome as early as 24–72 h after ROSC.^29^, ^30^, ^33^, ^34^, ^36^ NfL predicts neurological outcome after cardiac arrest at least as well as NSE.^33^, ^34^, ^37^ However, current guidelines do not yet recommend NfL for routine neuroprognostication, because consistent poor-outcome thresholds are lacking.^7^ Third, because up to half of patients may die before the 72nd hour and be non-evaluable, the informative sample may be smaller than the number randomised. Fourth, the trial is open-label for the treating team, since the intervention requires inflating or not the cuff. This is mitigated by allocation concealment through the electronic case report form, by an identical sham procedure that blinds patients and relatives, by blinded biomarker assays (except NSE as noted), and by a blinded mRS assessment at day 90. Finally, no interim analysis or data safety monitoring board is planned, justified by the procedure’s excellent tolerance^22^, ^26^ but precluding formal stopping rules.

### Trial status

The first patient was enrolled on 11 January 2025. The current version of the study protocol is V2.0, dated 4 May 2026. The estimated date of study completion is December 2027.

## Conclusion

RIPOST-CA is the first randomised controlled trial to evaluate whether early remote ischemic post-conditioning can attenuate ischaemia–reperfusion brain injury after OHCA. As a single-centre, exploratory trial, it is designed to provide the first biological and clinical evidence for this simple, non-invasive and inexpensive intervention. If the primary hypothesis is confirmed, RIPOST-CA would justify larger multicentre trials to assess the clinical benefit of remote ischemic post-conditioning and would help refine their design.

## Patient consent for publication

Not applicable.

## Declaration of generative AI in scientific writing

Artificial intelligence–based tools were used solely to assist with English language editing and proofreading of the manuscript. The authors take full responsibility for the scientific content and conclusions presented in this article.

## CRediT authorship contribution statement

**Jonathan Chelly:** Writing – original draft, Visualization, Supervision, Methodology, Investigation, Conceptualization. **Romain Cheyssac:** Writing – review & editing, Investigation, Conceptualization. **Thibaut Bertrand:** Writing – review & editing, Investigation. **Vincent Gardan:** Writing – review & editing, Software, Resources, Investigation, Data curation. **Asmaa Jobic:** Writing – review & editing, Supervision, Resources, Project administration, Methodology, Funding acquisition. **Masoud Rahmati:** Writing – original draft, Methodology, Formal analysis. **Romain Lambert:** Writing – review & editing, Investigation. **Julie Durif:** Writing – review & editing, Validation, Resources. **Vincent Sapin:** Writing – review & editing, Validation, Resources. **Alain Cariou:** Writing – review & editing, Methodology, Conceptualization. **Armel Dell Aria:** Writing – review & editing, Validation, Resources, Conceptualization. **Laurent Ducros:** Writing – review & editing, Validation, Investigation.

## Funding

This trial is supported by an extra-university research grant from the French Intensive Care Society (FICS) and by internal funding from the Centre Hospitalier Intercommunal Toulon La Seyne sur Mer.

## Declaration of competing interest

The authors declare the following financial interests/personal relationships which may be considered as potential competing interests: Jonathan Chelly reports financial support was provided by French Intensive Care Society. If there are other authors, they declare that they have no known competing financial interests or personal relationships that could have appeared to influence the work reported in this paper.

## Appendix A Supplementary material

Supplementary Data 1 SPIRIT 2025 editable checklist.

Supplementary Data 2 CONSORT 2025 editable checklist.

Supplementary Data 3 RIPOST-CA_Protocol_V2.0.
