# Supplementary material for: Neuroprotective effect of early remote ischaemic post-conditioning after out-of-hospital cardiac arrest (RIPOST-CA): study protocol for a randomised, single-blinded, monocentric, controlled trial
Source: Resusc Plus. 2026 Sep 24;32:101503. doi: 10.1016/j.resplu.2026.101503 (PMC13673021; doi:10.1016/j.resplu.2026.101503)
Supplement: Supplementary file 3 [file mmc3.docx]

| **CLINICAL RESEARCH PROTOCOL** | | |
| --- | --- | --- |

Neuroprotective effect of early remote ischaemic post-conditioning after out-of-hospital cardiac arrest: a single-centre, randomised, controlled, single-blinded trial

**– RIPOST-CA –**

**RIPOST-CA**

Protocol number: 2023-CHITS-010

IdRCB number: 2023-A02457-38

**Version 2.0 of 4 May 2026**

**Sponsor and data controller**

**Centre Hospitalier Intercommunal de Toulon La Seyne sur Mer (CHITS)**

**Clinical Research and Innovation Department (DRCI), GHT Var 83**

Rue Sainte Claire Deville, 83056 Toulon Cedex, France

Telephone: +33 (0)4 94 14 53 00 – Fax: +33 (0)4 94 14 53 01

**Project lead / Coordinating investigator**

**Pr Jonathan CHELLY**

Intensive Care Unit – CHITS – Hôpital Sainte Musse

54 rue Henri Sainte Claire Deville, 83100 Toulon, France

Telephone: +33 (0)4 94 14 51 24 – Fax: +33 (0)4 94 14 52 26

Email: jonathan.chelly@ch-toulon.fr

**RESEARCH CONTACTS**

| **Sponsor** | **Centre Hospitalier Intercommunal de Toulon La Seyne sur Mer (CHITS)**  Contact: Clinical Research and Innovation Department (DRCI) – Sponsorship Unit  Hôpital Sainte Musse  54 rue Henri Sainte Claire Deville, 83056 Toulon Cedex, France  Telephone (secretariat): +33 (0)4 94 14 55 25  Fax: +33 (0)4 94 14 55 26 |
| --- | --- |
| **Coordinating investigator / project lead** | **Pr Jonathan CHELLY**  Telephone: +33 (0)4 94 14 51 24  Email: jonathan.chelly@ch-toulon.fr |
| **Principal investigators** | **Pr Jonathan CHELLY** (Intensive Care, CHITS), **Dr Romain CHEYSSAC** (SAMU 83, CHITS) |
| **Data controller** | Centre Hospitalier Intercommunal de Toulon La Seyne sur Mer (CHITS)  Contact details of the CHITS data protection officer: dpo@ch-toulon.fr |
| **Structure responsible for project management and monitoring** | Clinical Research and Innovation Department (DRCI) – Sponsorship Unit  Centre Hospitalier Intercommunal de Toulon La Seyne sur Mer (CHITS) |
| **Structure responsible for methodology and statistics** | Clinical Research and Innovation Department (DRCI) – Sponsorship Unit  Centre Hospitalier Intercommunal de Toulon La Seyne sur Mer (CHITS) |
| **Structure responsible for data management and the database** | Clinical Research and Innovation Department (DRCI) – Sponsorship Unit  Centre Hospitalier Intercommunal de Toulon La Seyne sur Mer (CHITS) |
| **Investigating centres** | Two recruiting teams at CHITS:   - The Emergency Medical Service (SAMU) of the Var department (83); - The Polyvalent Intensive Care Unit team (patients admitted within 4 h of OHCA). |
| **Central laboratories** | - CHITS Medical Biology Laboratory: **Dr Armel DELL ARIA** - Medical Biology Laboratory, CHU de Clermont-Ferrand |
| **Associated research network providing scientific and methodological expertise** | AfterROSC network (https://afterrosc.org/) |

# 1 SYNOPSIS

Extract from protocol version 2.0 of 4 May 2026

IdRCB number: 2023-A02457-38

| **Title** | Neuroprotective effect of early remote ischaemic post-conditioning after out-of-hospital cardiac arrest: a single-centre, randomised, controlled, open-label trial. |
| --- | --- |
| **Acronym** | RIPOST-CA |
| **Research category** | RIPH 2 |
| **Coordinating investigator** | Pr Jonathan CHELLY |
| **Sponsor** | Centre Hospitalier Intercommunal de Toulon La Seyne sur Mer (CHITS) |
| **Rationale** | Patients admitted to the intensive care unit (ICU) after out-of-hospital cardiac arrest (OHCA) have high morbidity and mortality, mainly related to hypoxic-ischaemic brain injury. This injury may cause major neurological sequelae that can lead to a vegetative state and to the patient’s death following a decision to withdraw life-sustaining therapies or may progress to brain death.  This brain injury is mainly related to the ischaemia-reperfusion syndrome that is inseparable from the resuscitation of OHCA. This syndrome is responsible for a multi-organ inflammatory response and for the circulation of cytotoxic compounds throughout the body, including the brain.  Apart from standard intensive care measures (restoration of haemodynamic status, maintenance of cerebral homeostasis, prevention of systemic secondary brain insults), no specific therapy has so far improved the neurological prognosis of patients resuscitated from OHCA and admitted alive to the ICU.  Ischaemic conditioning consists of inducing short periods of ischaemia/reperfusion in a limb (arm or calf) by performing several alternating inflation/deflation cycles of a pneumatic tourniquet. Although the physiological mechanisms of this type of conditioning are not fully understood, several animal and human studies have demonstrated its protective effect against ischaemia-reperfusion injury, through the activation of humoral, neural and anti-inflammatory mechanisms. Human studies have already shown its benefit in myocardial infarction on the recovery of myocardial function, in ischaemic stroke with a reduction in neurological sequelae, and in septic shock with a reduction in mortality. Regarding cardiac arrest, preclinical studies in animal models have shown improved myocardial recovery and neurological outcome in subjects treated with ischaemic conditioning performed during or immediately after cardiopulmonary resuscitation (remote ischaemic post-conditioning, RIC).  The aim of our study is to demonstrate the benefit of early RIC, compared with standard care alone, in patients resuscitated from OHCA, on the reduction of neurological injury and organ failure related to the ischaemia-reperfusion syndrome. |
| **Primary objective** | To assess the impact of early RIC on the reduction of post-OHCA ischaemia-reperfusion axonal brain injury. |
| **Secondary objectives** | To assess the impact of early RIC in patients resuscitated from OHCA on:  1) The reduction of organ failure during the first three days after OHCA;  2) The proportion of patients with axonal brain injury considered severe;  3) The reduction of neuronal brain injury, and the proportion of patients with neuronal injury considered severe;  4) The reduction of astrocytic brain injury;  5) The reduction of mortality at ICU discharge;  6) The reduction in the number of patients with poor neurological outcome at ICU discharge and at 90 days after OHCA;  7) The occurrence of post-OHCA complications. |
| **Primary endpoint** | Comparison of the change in blood neurofilament light chain (NfL) levels, measured between the 6th and the 48th or 72nd hour after OHCA, if the patient has not died or been discharged from the ICU (samples taken at the 6th, 24th, 48th and 72nd hour after OHCA). If a patient dies before the 48th hour, he or she will be excluded from the analysis. If a patient dies between the 48th and 72nd hour, NfL measurements up to the 48th hour will be taken into account. |
| **Secondary endpoints** | Comparison between the control group and the intervention group of the following parameters:  1) The daily total Sequential Organ Failure Assessment (SOFA) score from day 1 to day 3 after OHCA and the SOFA score per organ from day 1 to day 3 after OHCA;  2) The proportion of patients with peak NfL > 500 pg/mL;  3) The change in NSE levels measured from the 6th to the 48th or 72nd hour after OHCA, and the proportion of patients with peak NSE > 60 µg/L;  4) The change in S100B levels measured from the 6th to the 48th or 72nd hour after OHCA;  5) The rate of ICU death related to multi-organ failure and the rate of ICU death related to neurological failure;  6) The proportion of patients with a modified Rankin Scale (mRS) score between 0 and 3 at ICU discharge and at 90 days after OHCA;  7) Comparison of the incidence of post-OHCA events (unexpected cardiac arrest in the ICU, iatrogenic event, limb ischaemia, bowel obstruction, mesenteric ischaemia, thromboembolic complication). |
| **Study population** | Patients who have had an OHCA and are on invasive mechanical ventilation for coma with a Glasgow Coma Scale score < 8. |
| **Inclusion criteria** | 1) OHCA regardless of the type of initial rhythm (shockable or non-shockable);  2) Sustained return of spontaneous circulation (ROSC) ≥ 20 minutes;  3) On invasive mechanical ventilation for coma (Glasgow < 8);  4) Availability of a lower limb without vascular access or blood pressure cuff in place;  5) Randomisation and application of the RIC procedure within a maximum of 4 hours after ROSC;  6) Consent of a relative or inclusion under the emergency procedure. |
| **Non-inclusion criteria** | 1) Age < 18 years, pregnant woman;  2) Patient unable to walk without assistance, unable to attend to own needs without assistance, bedridden, incontinent and requiring constant nursing care and attention (corresponding to an mRS of 4 or 5);  3) Estimated time between OHCA onset and ROSC (no-flow + low-flow) > 60 min;  4) Unwitnessed CA with initial asystole;  5) In-hospital CA;  6) Refractory CA (no ROSC considered sustained);  7) CA of traumatic or haemorrhagic origin, related to an acute brain condition (stroke, head injury), or related to hanging;  8) Persistent mean arterial pressure < 65 mmHg despite adequate fluid resuscitation and vasopressor and/or inotropic support;  9) Uncontrolled active haemorrhage;  10) Contraindication to, or impossibility of, placing a pneumatic tourniquet on either lower limb: bilateral lower-limb amputation; venous access in place on both lower limbs; pneumatic tourniquet size incompatible with the patient’s morphology;  11) Veno-arterial extracorporeal circulation (ECMO) initiated for refractory CA or refractory cardiogenic shock before inclusion;  12) Patient previously included in this study;  13) Inclusion in another interventional study targeting post-cardiac arrest prognosis;  14) Patient under a legal protection measure;  15) Patient without French social security coverage. |
| **Number of participants** | 48 patients (24 in each arm) |
| **Methodology** | Single-centre, interventional, balanced randomised, open-label study. |
| **Study conduct** | Patients with OHCA will be managed by prehospital emergency teams according to current guidelines (standard care). After resuscitation from OHCA and verification of the inclusion/non-inclusion criteria by the investigating physicians in charge of the patient, consent to participate will be sought from a relative of the patient. Inclusion may be performed by the investigating physician without consent if no relative can be contacted (inclusion under the emergency procedure), and consent to continue will then be sought subsequently (from relatives, then from the patient if his or her condition improves).  Inclusion and randomisation may be performed as early as the prehospital phase. If prehospital management did not allow inclusion, it may take place in the ICU provided the inclusion time limits are respected. In all cases, the patient must be included/randomised and the treatment arm applied within a maximum of 4 hours after OHCA. The primary endpoint will be assessed after the 48th or 72nd hour after OHCA if the patient has not died or been discharged from the ICU. If a patient dies before the 48th hour, he or she will be excluded from the analysis. If a patient dies between the 48th and 72nd hour, biomarker measurements up to the 48th hour will be considered.  Randomisation will be stratified by the OHCA rhythm (shockable or non-shockable).  The two study arms will be:  1) Intervention arm (standard care + RIC): same management as the control arm combined with three RIC sessions. The first session will be initiated as early as possible once the inclusion criteria are met, if possible, in the prehospital setting, otherwise on ICU admission, and at the latest within 4 hours after OHCA. The second and third sessions will be performed at the 12th and 24th hour after OHCA, respectively. One RIC session consists of inflating a pneumatic tourniquet on one thigh to 200 mmHg for 5 min, followed by deflation for 5 min, repeated 4 times in succession.  2) Control arm (standard care + “sham session”): patients managed in the prehospital setting and in the ICU according to current guidelines. A pneumatic tourniquet will also be placed at the same time points as described for the intervention group, without inflation, for the 40 minutes of each “sham session”.  Clinical and laboratory parameters will be recorded up to the 72nd hour after OHCA. Patients’ vital status will be assessed at ICU discharge, together with the cause of death where applicable. The mRS score will be assessed at ICU discharge and then at 90 days after OHCA. This information will be collected either directly during the hospital stay if the patient is still hospitalised, or by telephone from the patient or, where appropriate, from one of his or her relatives. |
| **Research timeline** | Maximum enrolment period: 36 months  Patient follow-up period: 3 months  Total study duration: 39 months |
| **Sources of funding** | SRLF extra-university grant 2023 (€15,000) + CHITS internal funding |
| **Independent Data Monitoring Committee planned** | No monitoring committee is planned, given:   - The very good tolerance of the procedure under investigation. Several studies that evaluated RIC in various settings (including after OHCA) reported no significant adverse effects provided that the precautions for use were respected (these are included in the non-inclusion criteria). - The absence of any planned interim analysis. |

# 2 SCHEDULE OF PARTICIPANT FOLLOW-UP

| **Visits** | **Baseline** | **H0** | **ICU admission** | **H6** | **H12** | **H24** | **H48** | **H72** | **ICU discharge** | **D90** |
| --- | --- | --- | --- | --- | --- | --- | --- | --- | --- | --- |
| Verification of eligibility criteria | X* |  |  |  |  |  |  |  |  |  |
| Information and collection of written consent, or inclusion in an emergency situation | X* |  |  |  |  |  |  |  |  |  |
| Randomisation |  | X* |  |  |  |  |  |  |  |  |
| Relevant medical history, epidemiological data (age, sex, weight, body mass index); CA history, Charlson score, Clinical Frailty Scale | X |  |  |  |  |  |  |  |  |  |
| Parameters at initial SAMU medical management | X |  |  |  |  |  |  |  |  |  |
| Application of the treatment arm (RIC or “sham session”) |  | No. 1* | |  | No. 2* | No. 3* |  |  |  |  |
| Aetiological investigations for the cause of CA (coronary angiography, angioplasty, etc.) | X |  |  |  |  |  |  |  |  |  |
| Parameters at ICU admission |  |  | X |  |  |  |  |  |  |  |
| SBP/DBP/MAP/HR/temperature |  | X | X | | | | | |  |  |
| Glasgow Coma Scale |  |  | X | | | | | |  |  |
| Arterial blood gases¹ |  |  | X |  |  | X | | |  |  |
| Use of RRT |  |  | X | | | | | | |  |
| Use of ECMO |  |  | X | | | | | | |  |
| Use of catecholamines |  | X | X | | | | | | |  |
| Laboratory tests² |  |  | X |  |  | X | X | X |  |  |
| NfL measurement |  |  |  | X* |  | X* | X* | X* |  |  |
| NSE measurement |  |  |  | X* |  | X | X | X |  |  |
| S100B measurement |  |  |  | X* |  | X* | X* | X* |  |  |
| SAPS II score |  |  |  |  |  | X |  |  |  |  |
| SOFA score |  |  |  |  |  | X | X | X |  |  |
| Vital status |  |  |  | X | X | X | X | X | X | X* |
| mRS assessment | X |  |  |  |  |  |  |  | X | X* |
| Post-OHCA complications |  | X | | | | | | |  |  |

¹ Arterial blood gases: pH, PaCO_2_, PaO_2_/FiO_2_, bicarbonate, lactate.

² Laboratory tests: platelets, creatinine, total bilirubin (+ at ICU admission only: leucocytes, prothrombin time, urea, CRP, troponin, procalcitonin).

* Performed for research purposes.

# 3 SCIENTIFIC RATIONALE AND JUSTIFICATION FOR THE RESEARCH

## 3.1 Background

Patients admitted to the intensive care unit (ICU) after a cardiac arrest (CA) occurring out of hospital (OHCA) have high morbidity and mortality, mainly related to hypoxic-ischaemic brain injury. Approximately two-thirds of ICU deaths after resuscitated OHCA are due to this brain injury **(1)**, which is mainly related to the ischaemia-reperfusion syndrome (IRS) as described by Negovsky in 1988 **(2)**. Ischaemia corresponds to the period between the onset of CA and the start of chest compressions, or “no-flow” period. Reperfusion corresponds to the period between the start of chest compressions and sustained return of spontaneous circulation (ROSC), corresponding to the “low flow” period, and extends into the first hours after CA. This IRS is responsible for a multi-organ inflammatory response and for the circulation of species that are toxic to cells throughout the body, including the brain **(3)**.

Apart from the standard post-CA intensive care measures recommended by scientific societies (restoration of haemodynamic status, maintenance of cerebral homeostasis, prevention of systemic secondary brain insults) **(4)**, no drug treatment or specific therapeutic procedure has so far reduced the organ injury, and particularly the brain injury, related to IRS. Reducing ischaemia-reperfusion injury therefore remains a major research target to improve the prognosis of CA patients.

Remote ischaemic post-conditioning (RIC) consists of inducing short periods of ischaemia-reperfusion in a limb, by performing several alternating inflation/deflation cycles with a blood pressure cuff, after the occurrence of the pathological event. Although the physiological mechanisms of action of RIC have not been fully identified, preclinical studies have demonstrated a humoral, anti-inflammatory and neural response underlying a cellular protective effect on all organs, including the brain **(5)**, of this intervention, which is simple to perform, non-invasive and inexpensive.

Inhibition of the mitochondrial permeability transition pore (mPTP) is one of the protective physiological mechanisms identified during RIC **(addendum no. 1)**, particularly when it is performed early after the occurrence of the pathological event **(6)**. Indeed, cell death induced during IRS is mediated in part by the opening of the mPTP.

In humans, studies have reported promising results in terms of morbidity and mortality with the use of RIC in several conditions: cardiovascular diseases **(7–10)**, ischaemic stroke **(11–13)**, severe traumatic brain injury **(14)** and septic shock **(15)**.

Regarding CA, preclinical studies in animal models have shown a reduction in ischaemic brain injury and an improvement in myocardial recovery and neurological outcome in subjects treated with ischaemic conditioning performed during or immediately after cardiopulmonary resuscitation **(16,17)**. A first, recently published study demonstrated the feasibility and safety of RIC performed as early as the prehospital phase in patients with OHCA **(18)**.

The primary objective of our study is to evaluate the benefit of early RIC in patients resuscitated from OHCA, compared with standard care alone, on the reduction of axonal neurological injury. We also wish to evaluate the benefit of RIC on the reduction of organ failure, the reduction of neuronal and glial neurological injury, and the improvement of post-CA neurological outcome.

## 3.2 Justification of the public interest of the research

OHCA remains a major public health problem, accounting for 275,000 and 350,000 sudden deaths each year in Europe and the United States, respectively **(19,20)**. In France, the most recent published data report 40,000 sudden deaths per year, with only 35% of patients admitted alive to the ICU **(21)**.

Despite improvements in prehospital care and the standardisation of basic and advanced cardiopulmonary resuscitation *via* the dissemination of international guidelines, the prognosis of patients with OHCA remains poor. Survival to hospital discharge after OHCA ranges from 2% for asystolic CA to 30% for CA with an initial shockable rhythm **(21,22)**. Only 10% of patients admitted alive to the ICU after resuscitated OHCA will leave hospital alive with few or no neurological sequelae **(23)**.

As stated above, no specific treatment or procedure has so far improved the vital and neurological prognosis of patients admitted to the ICU after resuscitated OHCA. The proposed study on RIC will provide a first clinical and biological evaluation of a non-invasive therapeutic procedure that is easy to implement (including as early as the prehospital phase **(18)**), low-risk and inexpensive, whose physiological mechanisms could have a significant brain-protective effect, and therefore potentially a beneficial effect on neurological outcome in this type of patient.

To date, few studies have been published in humans on the use of RIC after resuscitated OHCA. A first single-centre study in 30 patients with resuscitated OHCA demonstrated the feasibility and safety of a RIC procedure **(18)**. Another single-centre study was completed in 2021 in South Korea (registered on ClinicalTrials.gov under number NCT03093948), but its data have not yet been published. It aimed to evaluate the effect of one RIC session applied within 6 hours after resuscitated OHCA in patients admitted alive to hospital on the reduction of neuronal injury, assessed by blood neuron-specific enolase (NSE) at 48 hours after CA.

There is currently no study equivalent to the one we propose.

**The novel aspects of our study are:**

- The earliest possible delivery of RIC, to optimise the effect of this procedure on IRS and to achieve the most effective neuroprotection possible. Although we have defined a maximum time of 4 hours between sustained return of spontaneous circulation (ROSC) and randomisation, the possibility of performing inclusion, randomisation and the first RIC session as early as the prehospital phase should allow a shorter time to delivery, which will be monitored.
- A higher treatment “dose”, since we plan to perform 3 RIC sessions during the first 24 hours in order to maximise the cellular protective effect. This schedule of 3 sessions over 24 hours is similar to that of the RECO-Sepsis study, which showed a mortality benefit of RIC in patients with septic shock without any notable adverse effect **(15)**.
- In addition, hypoxic-ischaemic brain injury and the potential impact of RIC will be comprehensively assessed through the monitoring of blood levels of biomarkers specific to anatomically distinct injuries: axonal for neurofilament light chain (NfL), neuronal for NSE, and astrocytic for S100B protein (S100B).
- Finally, our study includes both a biological and a clinical evaluation, with monitoring of organ failure (assessed by the Sequential Organ Failure Assessment (SOFA) score, detailed in **addendum no. 2**), vital status and the patient’s neurological outcome up to 90 days after OHCA.

If our main hypothesis is confirmed, this study could justify further multicentre studies to evaluate the clinical benefit of RIC in this indication and could ultimately impact the management and outcome of patients resuscitated from OHCA.

## 3.3 Research objectives and hypotheses

The primary objective of this research is to evaluate the effect of early-initiated RIC on the reduction of hypoxic-ischaemic brain injury in patients resuscitated from OHCA. The main hypothesis is that RIC could reduce this hypoxic-ischaemic brain injury.

This study will therefore assess the severity of post-OHCA brain injury through the blood measurement of several validated brain biomarkers specific to axonal, neuronal and astrocytic injury: NfL, NSE and S100B **(24)**, respectively. As the axonal biomarker (NfL) has the most favourable sensitivity/specificity profile with respect to the severity of post-CA injury, it will be used as the primary endpoint **(25,26)**. The other biomarkers will be secondary endpoints.

## 3.4 Description of the intervention, administration schedule and duration of treatment

The experimental design of the study is detailed below:

**Experimental design of the RIPOST-CA study** (OHCA: out-of-hospital cardiac arrest; ROSC: sustained return of spontaneous circulation; RIC: remote ischaemic post-conditioning; SOFA: Sequential Organ Failure Assessment; mRS: modified Rankin Scale; NfL: neurofilament light chain; NSE: neuron-specific enolase; S100B: S100B protein).

Patients with OHCA will be managed by prehospital emergency teams according to current guidelines (standard care). After resuscitation from OHCA and verification of the inclusion/non-inclusion criteria by the investigating physicians in charge of the patient, consent to participate will be sought from a relative of the patient.

Given the study design, the inclusion criteria and the short inclusion time window required to assess the primary endpoint, inclusion under the emergency procedure may be performed by the investigating physician if no relative can be reached or contacted, and consent to continue will then be proposed subsequently (to relatives, then to the patient if his or her condition improves).

In order to benefit from the maximum potential effect of RIC in this indication, investigators will be encouraged to perform inclusion and randomisation and to apply the treatment arm as soon as possible after the inclusion criteria are met and after obtaining the consent of a relative (or, where applicable, after performing the emergency inclusion procedure). Inclusion may thus also be performed as early as the prehospital phase by an investigating physician. If prehospital management did not allow inclusion, it may take place in the ICU provided the inclusion time limits are respected. In all cases, the patient must be included/randomised and the treatment arm applied within a maximum of 4 hours after OHCA. The primary endpoint will be assessed after the 48th or 72nd hour after OHCA if the patient has not died or been discharged from the ICU. If a patient dies before the 48th hour, he or she will be excluded from the analysis. If a patient dies between the 48th and 72nd hour, biomarker measurements up to the 48th hour will be considered.

The two study arms will be:

1.	**Intervention arm (standard care + RIC):** same management as the control arm combined with three RIC sessions. The first session will be initiated as early as possible once the inclusion criteria are met, and at the latest within 4 hours after OHCA. The second and third RIC sessions will be performed at the 12th and 24th hour after OHCA, respectively. One RIC session consists of inflating a pneumatic tourniquet on one thigh to 200 mmHg for 5 min, followed by complete deflation for 5 min, repeated 4 times in succession.

2.	**Control arm (standard care + “sham session”):** patients managed in the prehospital setting and in the ICU according to current guidelines. A pneumatic tourniquet will also be placed and kept continuously deflated for 40 minutes, corresponding to a first “sham session”. The second and third “sham sessions” will be performed at the 12th and 24th hour after OHCA, respectively.

Randomisation will be stratified by the OHCA rhythm (shockable or non-shockable).

Clinical and laboratory parameters will be recorded up to the 72nd hour after OHCA. Patients’ vital status will be assessed at ICU discharge, together with the mode of death where applicable. Neurological outcome will be assessed using the modified Rankin Scale (mRS, detailed in **addendum no. 3**) at ICU discharge and then at 90 days after OHCA. This information will be collected either directly during the hospital stay if the patient is still hospitalised, or by telephone from the patient or, where appropriate, from one of his or her relatives.

## 3.5 Benefit/risk assessment

### 3.5.1 Benefits

If the study is completed and our main hypothesis is confirmed, i.e. a reduction in post-OHCA hypoxic-ischaemic injury in patients receiving RIC sessions, the potential benefit for patients in terms of morbidity and mortality could be substantial.

Indeed, the results of this study could justify larger studies to evaluate the clinical benefit of this procedure, with improved survival with good neurological outcome as the primary objective.

The results of this study will also help guide the design and conduct of a future multicentre randomised controlled trial (sample size calculation, refinement of protocol procedures, stratification and identification of patient subgroups, completeness of data collection, etc.).

### 3.5.2 Risks

This study involves minimal risks and burdens. We are evaluating RIC, a non-drug therapeutic procedure, which therefore falls within the scope of a category 2 research project involving human participants (RIPH 2).

In accordance with an RIPH 2 project, RIC consists of an external mechanical stimulation performed with a pneumatic tourniquet, a medical device used in accordance with its CE marking.

It should also be noted that RIC has been studied in several critical conditions requiring intensive or critical care (myocardial infarction, cardiac surgery, ischaemic stroke, severe traumatic brain injury, septic shock). A first clinical study in post-OHCA patients also confirmed the feasibility and safety of RIC in this type of patient **(18)**. All studies have shown excellent clinical tolerance of RIC sessions and reported no notable side effects, provided that the contraindications to this type of procedure were respected (these are included in the study’s exclusion criteria).

The theoretical risks of RIC are rare but may include:

- Temporary worsening of pre-existing ischaemic lesions in the limb used for RIC.
- Transient arterial hypotension related to blood pooling during tourniquet inflation, reversible upon deflation.
- Occlusion of a vascular access line if the procedure is performed on the limb where it is placed.

The inclusion and non-inclusion criteria of the study drastically reduce the risk of such complications. Furthermore, in the event of hypotension at the scheduled time of a RIC session, the session will be postponed for the time needed to stabilise the patient’s haemodynamic status with usual therapies (fluid resuscitation, initiation/increase of catecholamines).

In addition, RIC has no theoretical contraindication during pregnancy. However, the precautionary principle and the absence of literature on the subject led us to exclude pregnant women from the study. The maximum inclusion time window of 4 hours allows pregnancy to be ruled out before inclusion in the event of OHCA in an eligible patient of childbearing age.

# 4 OBJECTIVES AND ENDPOINTS

## 4.1 Primary objective

The primary objective of the RIPOST-CA study is to assess the impact of early RIC on the reduction of ischaemia-reperfusion axonal brain injury in patients resuscitated from OHCA.

## 4.2 Primary endpoint

The primary endpoint will be the change in blood neurofilament light chain (NfL) levels, measured between the 6th and the 48th or 72nd hour after OHCA if the patient has not died or been discharged from the ICU (samples taken at the 6th, 24th, 48th and 72nd hour after OHCA). If a patient dies before the 48th hour, he or she will be excluded from the analysis. If a patient dies between the 48th and 72nd hour, NfL measurements up to the 48th hour will be considered.

## 4.3 Secondary objectives

The secondary objectives are to assess the impact of early RIC in patients resuscitated from OHCA on:

1.	The reduction of organ failure during the first three days after OHCA;

2.	The proportion of patients with axonal brain injury considered severe;

3.	The reduction of neuronal brain injury, and the proportion of patients with neuronal injury considered severe;

4.	The reduction of astrocytic brain injury;

5.	The reduction of mortality at ICU discharge;

6.	The reduction in the number of patients with poor neurological outcome at ICU discharge and at 90 days after OHCA;

7.	The occurrence of post-OHCA complications.

## 4.4 Secondary endpoints

The following secondary endpoints will be compared between the two arms:

1.	The daily total SOFA score from day 1 to day 3 after OHCA (in the event of death within the first three days, the SOFA score will be considered maximal, i.e. 24 points); the SOFA score per organ from day 1 to day 3 after OHCA (in the event of death within the first three days, the maximal per-organ SOFA score will be assigned);

2.	The proportion of patients with peak NfL > 500 pg/mL;

3.	The change in NSE levels measured from the 6th to the 48th or 72nd hour after OHCA if the patient has not died or been discharged from the ICU (samples taken at the 6th, 24th, 48th and 72nd hour after OHCA), and the proportion of patients with peak NSE > 60 µg/L;

4.	The change in S100B levels measured from the 6th to the 48th or 72nd hour after OHCA if the patient has not died or been discharged from the ICU (samples taken at the 6th, 24th, 48th and 72nd hour after OHCA);

5.	The rate of ICU death related to multi-organ failure and the rate of ICU death related to neurological failure;

6.	The proportion of patients with a favourable neurological outcome at ICU discharge and at 90 days after OHCA. A favourable outcome is defined as an mRS score between 0 and 3;

7.	The proportion of patients with a post-OHCA complication (unexpected cardiac arrest in the ICU, iatrogenic event, limb ischaemia, bowel obstruction, mesenteric ischaemia, thromboembolic complication) and the number of complications per patient.

# 5 METHODOLOGY

## 5.1 Type of research

This is an interventional study evaluating a procedure (RIC) involving minimal risks and burdens (RIPH 2), consisting of an external mechanical stimulation performed with a pneumatic tourniquet, a medical device used in accordance with its CE marking.

The study is a prospective, single-centre, comparative, randomised, open-label study.

## 5.2 Randomisation

Patients meeting all the eligibility criteria of the research will be randomised (after inclusion under the emergency procedure or after collection of a relative’s consent – **see section 7.2**) in a 1:1 ratio between the following two groups:

- Control arm: post-OHCA management according to current guidelines + three “sham sessions”.
- Intervention arm: post-OHCA management according to current guidelines + three RIC sessions.

Randomisation using permuted blocks of random sizes will be stratified by the OHCA rhythm (shockable or non-shockable).

The randomisation list will be generated by the statistician, under the responsibility of the sponsor, before the start of the research and will be kept confidential in a secure location. It will then be implemented in an eCRF.

Randomisation will be performed by the investigating centre team via the eCRF at the time of inclusion.

## 5.3 Number of participating centres

This is a single-centre study conducted at the Centre Hospitalier Intercommunal de Toulon La Seyne-sur-Mer (CHITS).

The teams involved in the study will be:

- The team of the Emergency Medical Service (SAMU) of the Var department (83) and of the Toulon Mobile Emergency and Intensive Care Unit (SMUR), based at CHITS (recruiting centre).
- The team of the CHITS Polyvalent Intensive Care Unit (recruiting centre).

## 5.4 Research timeline and expected duration of participation

The enrolment period is 36 months. The duration of the evaluated treatment is 24 hours and the follow-up period of included participants is 3 months.

The end of the research will be defined as the end of participation of the last person taking part in the research. The total duration of the research will be 39 months.

# 6 STUDY POPULATION

## 6.1 Inclusion criteria

The inclusion criteria are:

1.	OHCA regardless of the type of rhythm (shockable or non-shockable);

2.	Sustained return of spontaneous circulation (ROSC) ≥ 20 minutes;

3.	On invasive mechanical ventilation for post-ROSC coma (Glasgow < 8 – score detailed in **addendum no. 4**);

4.	Availability of a lower limb without vascular access or blood pressure cuff in place;

5.	Randomisation and application of the first RIC session (or “sham session”) within a maximum of 4 hours after ROSC;

6.	Consent of a relative or inclusion under the emergency procedure.

## 6.2 Non-inclusion criteria

The non-inclusion criteria are:

1.	Age < 18 years, pregnant woman;

2.	Patient unable to walk without assistance, unable to attend to own needs without assistance, bedridden, incontinent and requiring constant nursing care and attention (corresponding to an mRS of 4 or 5);

3.	Time between OHCA onset and ROSC (“no-flow” + “low-flow”) > 60 min;

4.	Unwitnessed OHCA with initial asystole;

5.	Refractory OHCA (no ROSC considered sustained);

6.	OHCA of traumatic or haemorrhagic origin, related to an evident acute brain condition (stroke, head injury), or related to hanging;

7.	In-hospital CA;

8.	Persistent mean arterial pressure < 65 mmHg despite adequate fluid resuscitation and vasopressor and/or inotropic support;

9.	Uncontrolled active haemorrhage;

10.	Contraindication to, or impossibility of, placing a pneumatic tourniquet on either lower limb:

- Bilateral lower-limb amputation
- Venous access in place on both lower limbs
- Pneumatic tourniquet size incompatible with the patient’s morphology

11.	Veno-arterial extracorporeal circulation (ECMO) initiated for refractory cardiogenic shock before inclusion;

12.	Patient previously included in this study;

13.	Inclusion in another interventional study targeting post-cardiac arrest prognosis;

14.	Patient under a legal protection measure;

15.	Patient without French social security coverage.

# 7 STUDY PROCEDURES

## 7.1 Recruitment

The study population consists of patients who have had an out-of-hospital cardiac arrest (OHCA) with sustained resuscitation (ROSC > 20 minutes) and whose admission to the intensive care unit of the Centre Hospitalier Intercommunal de Toulon La Seyne-sur-Mer has been arranged with SAMU 83.

Patients will be jointly identified by the investigating physicians of the Centre Hospitalier Intercommunal de Toulon La Seyne-sur-Mer (physician of the prehospital team managing the patient, dispatching physician referring the patient to the ICU, physician managing the patient in the ICU).

## 7.2 Information and consent procedures for persons taking part in the research

| **Persons whose consent is sought** | **Who informs and obtains consent** | **When the person is informed** | **When the person’s consent is obtained** |
| --- | --- | --- | --- |
| A family member  **THEN**  The person taking part in the research | The investigator  **OR**  His/her representative (collaborator of the investigator) | The family member(s) will be informed as soon as they can be reached, as early as possible.  If the patient’s condition improves, the patient will be informed upon awakening, once his/her cognitive state is compatible with receiving informed information. | After a sufficient reflection period and after having received informed information. |

In accordance with Article L1122-1-1 of the French Public Health Code, no research involving human participants may be carried out on a person without his or her free and informed consent, obtained in writing after information about the study has been provided.

However, in the case of research involving human participants to be carried out in emergency situations that do not allow the prior consent of the person concerned to be obtained, where such consent is required, the protocol submitted for the opinion of the committee referred to in Article L1123-1 may provide that this person’s consent is not sought and that only the consent of his or her family members or of the trusted person referred to in Article L1111-6 is sought, under the conditions set out in Article L1122-1-1, if they are present. The protocol may provide for a derogation from this obligation in the case of an immediate life-threatening emergency, as assessed by this committee. The person concerned or, where applicable, the family members or the trusted person referred to in Article L1111-6 are informed as soon as possible and their consent, where required, is sought for the possible continuation of the research. They may also object to the use of data concerning the person in the context of this research.

Three features of our study justify the derogation allowing inclusion in an emergency situation:

1.	All eligible patients are, by definition, unable to express their consent (patients with OHCA and persistent coma thereafter).

2.	The relatives of patients with OHCA are not always identified or identifiable in the first hours or days of management.

3.	RIC must be initiated as early as possible to achieve the greatest possible effect on the reduction of hypoxic-ischaemic brain injury. We therefore plan inclusion within a maximum of four hours after resuscitation from OHCA.

Thus, in this study:

- Inclusion may be performed without the patient’s consent, provided that a relative of the patient is informed and his or her consent is obtained (Article L1122-2 of the CSP). Given the urgency of the situation, the relative will be given a minimum reflection period of one hour. This period may not extend beyond 4 hours after the cardiac arrest.
- In the absence of a relative (isolated patient or relative who cannot be reached in the first hours of management), inclusion in an emergency situation may be performed by the study investigating physician (Article L1122-1-3 of the CSP). As soon as possible, family members or the trusted person will be informed, and their consent will be sought for the possible continuation of the research.
- As soon as the patient is able to express free and informed consent, this consent must be obtained by the investigator in charge of the patient for continuation of the study and use of the data collected. If the patient remains unable to express consent, the consent of a relative must be obtained. After a sufficient reflection period, the physician will ensure that the patient or relative has a general understanding of the study and will obtain written consent to participate in the research. The free, informed and written consent of the person is obtained by the principal investigator or by a physician representing him or her.
- Outside the emergency situation, the relative or the patient will be given 24 hours to decide on continuation of the research.

A copy of the information sheet and consent form, dated and signed by the person taking part in the research or his or her relative and by the principal investigator or the physician representing him or her, will be given to the person. The principal investigator or the physician representing him or her will also keep a copy.

The investigator will also record in the patient’s medical file the patient’s participation in the research, the procedures used to obtain his or her consent or the consent of his or her relative, and the procedures used to provide the information.

## 7.3 Research visits

### 7.3.1 Inclusion visit (D0)

At the D0 visit, and if a relative is present or can be reached, the investigating physician begins by:

- Presenting the research as a whole;
- Answering the relative’s questions, particularly regarding the aim, conduct, constraints, risks and benefits of the research;
- Ensuring that the relative has had time to make a free decision, has been able to read the entire information and consent document and has understood it;
- Obtaining the relative’s signed informed consent;
- Recording the patient’s participation in the research in the medical file.

Once the relative’s consent has been obtained, the investigating physician may:

- Verify all inclusion and non-inclusion criteria;
- If all eligibility criteria are met, record in the medical file the patient code and the group (intervention or control) allocated by randomisation:

|__|__| / |__| / |__|

inclusion number (chronological order) / first letter of surname / first letter of first name

If no relatives are present when the patient is being managed, the investigating physician may proceed with emergency inclusion of the patient and randomisation. Consent to continue will be sought subsequently, either when a relative comes forward and/or when the patient awakens.

### 7.3.2 Follow-up visits

Patients will be followed for 3 days from inclusion, during hospitalisation and within the framework of the study. Vital status and an mRS assessment at ICU discharge will also be collected. A follow-up visit is scheduled at three months after the date of inclusion to collect vital status and a further mRS assessment.

This information will be collected either directly during the hospital stay if the patient is still hospitalised, or by telephone from the patient or, where appropriate, from the trusted person (designated at the start of the study).

### 7.3.3 End of research participation

All surviving patients will be contacted by telephone three months after inclusion. The patient’s participation in the research ends after this visit.

However, participation may end prematurely:

1.	By decision of the investigator;

2.	By decision of the patient;

3.	In the event of the patient’s death.

### 7.3.4 Exclusion period after the research

No exclusion period after the patient’s participation in this research is planned.

During his or her participation, the patient may not take part in another interventional research protocol.

## 7.4 Compensation of participants

No compensation of participants is planned in this study.

## 7.5 Distinction between routine care and research

|  | **Procedures associated with routine care** | **Procedures added by the research** |
| --- | --- | --- |
| **Consultations** | X | Telephone follow-up at 3 months (vital status, mRS) |
| **Treatments** | Post-OHCA management according to current guidelines | 3 RIC sessions or 3 “sham sessions” |
| **Examinations** | Aetiological work-up (coronary angiography, brain and chest CT scan, echocardiography), standard laboratory tests, prognostic assessment (NSE at H24-H48-H72, electroencephalogram, somatosensory evoked potentials, brain imaging, pupillometry). | Blood NfL measurements at H6, H24, H48 and H72 (4 mL per sample)  Blood NSE measurement at H6 (2 mL)  Blood S100B measurements at H6, H24, H48 and H72 (4 mL per sample) |
| **Other** | X | X |

## 7.6 Premature discontinuation of treatment and/or research

### 7.6.1 Discontinuation of the study by the sponsor

The sponsor may stop the study at any time for the following reasons:

- Inability of the investigator to include participants according to the planned schedule;
- Absence of signed consent;
- Major protocol violations;
- Incomplete or erroneous data.

The sponsor will submit an end-of-trial declaration within 90 days of the end of the research and will inform the French regulatory authorities.

### 7.6.2 Discontinuation of the study by the investigator

In the event of an adverse event considered severe by the investigator and potentially endangering the health of participants, the investigator may stop the patient’s participation in the research in agreement with the sponsor.

The investigator must:

- Document the reason(s);
- Collect the endpoints at the time of discontinuation, if the participant agrees;
- Arrange follow-up of the participant.

### 7.6.3 Criteria and procedures for premature discontinuation of a participant

Any participant, family member or legal representative may stop participation in the research at any time and for any reason.

If, during participation in the research, the participant experiences a serious adverse event requiring discontinuation of treatment, treatment must be stopped but the participant will continue to be followed in the research.

The investigator may temporarily or permanently interrupt a participant’s participation in the research for any reason affecting his or her safety or that would best serve the participant’s interests.

In the event of premature discontinuation of a participant or withdrawal of consent, data collected before discontinuation may be used. The investigator must document the date and reason for discontinuation in the participant’s source file and in the eCRF, which must list the different reasons for discontinuation of participation in the research:

- Lack of efficacy;
- Adverse event;
- Other medical problem;
- Personal reason of the participant;
- Explicit withdrawal of consent;
- Lost to follow-up.

### 7.6.4 Withdrawal of consent

A participant, family member or legal representative who wishes to withdraw consent to participate in the research, as they are entitled to do at any time, is no longer followed within the protocol but must receive the best possible care given his or her state of health and current knowledge.

When a participant withdraws consent to participate in the research, the investigator must notify the sponsor. From the date of withdrawal of consent, no further data may be collected. Any data concerning the participant collected after the date of withdrawal of consent must then be permanently deleted from the database, in accordance with applicable data protection legislation (French Data Protection Act No. 78-17 of 6 January 1978, as amended, and the General Data Protection Regulation).

The date of withdrawal of consent and the reasons for withdrawal must be documented in the eCRF and in the source file.

### 7.6.5 Participants lost to follow-up

When a participant does not respond to the telephone follow-up visit, the investigating centre must make every effort to re-establish contact with the person (and record this in the source file). During this contact, the information required by the protocol must be obtained as far as possible.

### 7.6.6 Replacement of participants, where applicable

Whatever the reason for discontinuation of a randomised participant (withdrawal from the study, lost to follow-up, etc.), he or she will not be replaced. If an included patient is withdrawn from the study, his or her number will not be reassigned.

### 7.6.7 Follow-up of participants withdrawn from the trial

Withdrawal of a patient from the research will in no way change his or her usual management of the condition.

# 8 END OF THE RESEARCH

## 8.1 Definition of the end of the research

The end of the research will be defined by the last visit of the last participant. At the end of the research or in the event of premature discontinuation of the study intervention, participants will be managed as usual within routine care.

## 8.2 Communication of research results

Participants who have expressed the wish will be informed of the overall results of the research by means of a letter written and submitted to the CPP for information, based on the summary of the final research report. This letter will be sent by the investigating centre. Its dispatch will be recorded in each participant’s source file.

# 9 RESEARCH COMMITTEES

## 9.1 Steering committee

A steering committee will be set up, consisting of the coordinating investigator, the scientific lead, the methodologists in charge of the project and a representative of the sponsor. This committee initiated the project and will write and validate the study documents. It will initially determine the methodology, decide during the research on the course of action in the event of unforeseen circumstances, and oversee the conduct of the research.

The steering committee may propose to extend or stop the study in the event of excessively slow enrolment, major protocol violations, or for medical and/or administrative reasons.

## 9.2 Independent Data Monitoring Committee

This RIPH 2 study does not require an Independent Data Monitoring Committee, given:

- The very good tolerance of the procedure under investigation: several studies have evaluated RIC and reported no significant adverse effects provided that the precautions for use were respected (included in the study’s non-inclusion criteria).
- The absence of planned interim analyses.

# 10 STATISTICAL METHODS AND SAMPLE SIZE CALCULATION

Statistical analyses will be performed by a biostatistician under the authority of the sponsor, using R (version 4.0.2 or later) or SAS® software (version 9.4 or later).

The justification of the number of patients required and a general description of the statistical methods to be used to analyse the data are presented below.

## 10.1 Sample size calculation

The sample size was determined based on the primary endpoint, i.e. the comparison between the two management arms of the change in blood NfL levels measured between the 6th and 72nd hour after OHCA.

The hypothesis test of this study is therefore designed to show a 50% reduction in NfL between the 6th and 72nd hour in the intervention arm compared with the control arm. Based on the studies by Moseby-Knappe et al. **(27)** and Adler et al. **(25)**, mean (standard deviation) NfL values at 72 h may be assumed to be 3,250 (2,000) pg/mL in the control arm and 1,625 (1,000) pg/mL in the intervention arm. With a type I error rate (alpha) of 5%, 16 patients per arm would be required to achieve a minimum statistical power of 80%. Stratified randomisation should ensure that NfL levels at 6 h are balanced between arms. The changes in NfL levels between the 6th and 72nd hour after OHCA in the two arms are therefore fully comparable to the 72-hour NfL levels estimated above for the purposes of the calculation.

Considering that 50% of included patients may die between ICU admission and the 72nd hour after OHCA (and would therefore not be evaluable for the primary endpoint), a minimum of 24 patients per group is planned, i.e. a total of 48 patients, over an enrolment period of 36 months.

## 10.2 Analysis populations

Three analysis populations are defined:

- **Full analysis set (FAS):** all randomised patients with valid informed consent. The FAS is used to describe the flow of participants and to describe and compare patient characteristics by randomised arm (intention-to-treat).
- **Modified intention-to-treat (mITT) population:** randomised patients surviving to the 48th hour after OHCA with at least one NfL value available between the 6th and the 72nd hour (or the 48th hour for patients dying between the 48th and 72nd hour), analysed according to the arm allocated by randomisation. The mITT population is used for the sensitivity analysis of the primary endpoint.
- **Per-protocol (PP) population:** patients who received the procedures allocated by randomisation without major protocol deviation and who are evaluable for the primary endpoint, i.e. with blood samples allowing quantification of NfL levels between the 6th and the 48th or 72nd hour after OHCA. The PP population is used for the primary and secondary analyses, consistent with the objective of estimating the biological efficacy of RIC as delivered. Major protocol deviations will be defined before database lock.

## 10.3 Timing of analyses

A single final analysis is planned for this research. No interim analysis is planned. The analyses of the primary and secondary endpoints will be performed once all included patients have completed their participation, i.e. 90 days after OHCA (or at death, if this occurs earlier).

## 10.4 Statistical analyses

### 10.4.1 General principles

Categorical variables will be described using frequencies and percentages. Continuous variables will be described using the number of observations, mean, standard deviation, median, and minimum and maximum values. Patient characteristics and other baseline data (demographics, disease characteristics, clinical and laboratory data) will be summarised to characterise the study population.

Missing values will be reported and will not be included in the calculation of proportions. Missing data will not be imputed, except for the pre-specified worst-case rule assigning the maximum SOFA score to patients who die during the first three days after OHCA (24 points for the total SOFA score and 4 points for each organ-specific score).

All tests will be two-sided, with a significance level of 0.05.

### 10.4.2 Disposition of patients in the study

A flow chart based on the FAS will graphically represent the progress of the research over time and will show the number of patients in each of the analysis populations defined above before any statistical analysis.

### 10.4.3 Patient characteristics

All data collected in this research will be described overall and compared according to the randomised arm in the FAS (intention-to-treat analysis).

### 10.4.4 Analysis of the primary endpoint

**Main analysis.** The change in NfL between the 6th and 72nd hour after OHCA (or the 48th hour for patients dying between the 48th and 72nd hour) and the peak NfL value over this interval will be compared between arms in the PP population using Wilcoxon–Mann–Whitney tests. The effect size will be estimated using the Hodges–Lehmann estimator of the median difference between arms, with its 95% confidence interval (CI).

**Longitudinal analysis.** Changes in NfL across sampling times (6th, 24th, 48th and 72nd hour) and between arms will be analysed using a linear mixed-effects model including arm, time and the arm-by-time interaction as fixed effects, a random intercept for patient, and adjustment for the randomisation stratification factor (initial rhythm: shockable or non-shockable). NfL values will be log-transformed before modelling to satisfy the normality assumption, which will be checked on model residuals using the Shapiro–Wilk test and QQ plots. This model accommodates incomplete follow-up without imputation. The arm-by-time interaction constitutes the primary test of a time-varying treatment effect. This analysis will be performed in the PP population.

**Sensitivity analysis.** The analyses described above (Wilcoxon–Mann–Whitney tests with Hodges–Lehmann estimates, and the linear mixed-effects model) will be repeated in the mITT population.

**Exploratory analyses.** Associations between NfL kinetics and the other biomarkers (NSE, S100B) or the SOFA score will be explored using Spearman correlation coefficients.

### 10.4.5 Analysis of secondary endpoints

Secondary endpoints will be analysed in the PP population.

- **Organ failure:** total and organ-specific SOFA scores at 24, 48 and 72 hours (with the worst-case rule described in section 10.4.1 for patients dying within the first three days) will be compared between arms using Wilcoxon–Mann–Whitney tests or linear mixed-effects models.
- **Neuronal and astrocytic injury:** NSE and S100B levels collected from the 6th to the 72nd hour after OHCA (daily values, peak and change over time) will be compared between arms using Wilcoxon–Mann–Whitney tests or linear mixed-effects models, as for the primary endpoint.
- **Binary endpoints:** the proportions of patients with peak NfL > 500 pg/mL and peak NSE > 60 µg/L, ICU death rates by cause (multi-organ failure, neurological failure), and the proportions of patients with a favourable neurological outcome (mRS 0–3) at ICU discharge and at day 90 will be compared using chi-square tests (or Fisher’s exact tests where appropriate). Odds ratios (OR) with 95% CI will be estimated by logistic regression adjusted for the randomisation stratification factor, with the Wald test used to assess the significance of the model coefficient.
- **Post-OHCA complications:** the proportion of patients with at least one post-OHCA complication will be compared using the chi-square test (or Fisher’s exact test), and the number of complications per patient using the Wilcoxon–Mann–Whitney test.

### 10.4.6 Multiplicity

Given their exploratory nature, secondary endpoints will not be adjusted for multiplicity and will be interpreted as hypothesis-generating.

# 11 QUALITY CONTROL AND ASSURANCE

## 11.1 General organisation

The sponsor must ensure the safety of, and respect for, the persons who have agreed to take part in the research. It must implement a quality assurance system allowing optimal monitoring of the conduct of the research at the investigating centres.

To this end, the sponsor appoints clinical research associates (CRAs), whose main task is to carry out regular monitoring visits at the research sites after performing the site initiation visits.

The objectives of research monitoring, as defined in Good Clinical Practice (GCP § 5.18.1), are to verify that:

- The rights, safety and protection of persons taking part in the research are respected;
- The reported data are accurate, complete and consistent with the source documents;
- The research is conducted in accordance with the current protocol, GCP and applicable legal and regulatory requirements.

## 11.2 Quality control

A CRA appointed by the sponsor will ensure the proper conduct of the research and of data collection, documentation, recording and reporting, in accordance with the standard operating procedures implemented by the sponsor and with GCP, as well as with applicable legal and regulatory requirements.

The investigator and the members of his or her team agree to make themselves available during the quality control visits carried out at regular intervals by the CRA. During these visits, the following will be reviewed:

- Collection of consent;
- Compliance with the research protocol and the procedures defined therein;
- Quality of the data collected in the case report form: accuracy, missing data, consistency of data with “source” documents (medical records, appointment books, original laboratory results, etc.).

## 11.3 Protocol deviations – management of non-conformities

Failure to comply with the protocol, the standard operating procedures of the protocol, GCP or applicable legal and regulatory requirements by an investigator, a member of the sponsor’s staff or any other person involved in the conduct of the research is qualified as a protocol deviation and must promptly lead to the implementation of appropriate measures.

Any event occurring because of failure to comply with the protocol, standard operating procedures, GCP or applicable legal and regulatory requirements by an investigator or any other person involved in the conduct of the research must be reported to the sponsor.

No derogation from the protocol will be authorised by the sponsor. The investigator undertakes to comply with the provisions of the protocol, particularly regarding the conditions for conducting the research and the procedures for expressing and publishing the results. However, if a protocol deviation occurs, the investigator, or any person he or she has designated, must justify it, document it and send the information to the sponsor as soon as possible.

Departures and deviations from the applicable standards are classified according to the sponsor’s internal procedures. Depending on the severity of the deviation, the sponsor will take appropriate measures. Depending on the deviation, the end of participation of the participant concerned may be considered.

All protocol deviations will be presented to the research steering committee.

If a violation, a major deviation or a deliberately repeated deviation by an investigator or another person involved in the research is detected during research monitoring, quality control or an audit, the sponsor may terminate that person’s participation in the research.

## 11.4 Audit – inspection

Investigators agree to accept quality assurance audits performed by the sponsor and inspections performed by the competent authorities. All data, documents and reports may be subject to audits and regulatory inspections, and medical confidentiality may not be invoked against them.

An audit may be carried out at any time by persons appointed by the sponsor who are independent of those responsible for the research. Its purpose is to ensure the quality of the research, the validity of its results and compliance with applicable laws and regulations.

The persons who manage and monitor the research agree to comply with the requirements of the sponsor and of the competent authority regarding an audit or inspection of the research.

The audit may apply to all stages of the research, from protocol development to publication of the results and filing of the data used or produced in the research. Each audit is conducted according to a procedure validated by the sponsor that defines the procedures, scope and standards of the audit.

The audit plan and procedures are established according to the importance and complexity of the research, the number of persons included and the foreseeable risks to participants. The auditor’s observations and findings are documented. For each audit, the sponsor and the audited party receive an audit certificate.

## 11.5 Investigators’ obligations

In accordance with GCP aimed at ensuring the quality of the research, each investigator undertakes to:

- Respect the rights of participants and ensure their safety and well-being;
- Ensure his or her availability and that of his or her team;
- Ensure that his or her recruitment capacity is compatible with the conduct of the research;
- Take responsibility for organising the technical facilities for setting up research-specific circuits (consultations, sampling), for keeping documents during the research and then for archiving and destroying data and documents during the regulatory period;
- Obtain and archive participants’ written consent in a secure place;
- Ensure compliance with the protocol and the quality of the data in the case report form, as well as their regular transmission to the sponsor within the recommended time frames;
- Allow regular monitoring of the research by a representative of the sponsor, who must have access to participants’ source documents to validate the data reported in the case report forms. The project manager or the principal investigator may be contacted at any time for any question relating to the protocol, its practical application or the course of action to be taken in the event of certain events;
- Accept any audit of the research directly by the sponsor or, with its authorisation, by other organisations;
- Accept inspection of the research by the authorised Health Authorities.

# 12 VIGILANCE

In accordance with Article L1123-10 of the French Public Health Code, for RIPH 2 research, “standard” vigilance rules apply to each practice under investigation in this research.

# 13 DATA MONITORING AND MANAGEMENT

## 13.1 Data confidentiality – participant code

During the research, individual data collected on the persons taking part in it and transmitted to the sponsor by the investigators (or any other qualified party) will be rendered non-identifying and must under no circumstances show in plain text the names of the persons, their addresses or any other information allowing their direct identification. They will remain strictly confidential and will be pseudonymised. To this end, each participant in the research will be assigned an identifier composed as follows:

|__|__| / |__| / |__|

inclusion number (chronological order) / first letter of surname / first letter of first name

This reference is unique and will be kept for the entire duration of the research. In order to respect the anonymity of persons taking part in the research, only this code will be recorded in the case report form.

## 13.2 Data collection procedures

Data are collected and validated under the responsibility of the principal investigator of each centre. The information required by the research protocol will be collected in the electronic case report form (eCRF). The research database will then be built from the data collected in the eCRF.

Any discrepant data in the eCRF will be the subject of a query. All changes to data in the eCRF will be traced.

Participant coding will be ensured using numbers, recorded on all documents required for the research, and by the removal of all identifying information from copies of source documents.

## 13.3 Right of access to data and source documents

In accordance with GCP:

- The sponsor is responsible for obtaining the agreement of all parties involved in the research to guarantee direct access to all research sites, source data, source documents and reports for the purposes of quality control and audit by the sponsor;
- Investigators will make available the documents and individual data strictly necessary for monitoring, quality control (in particular monitoring visits) and audit of the research to the persons responsible for the monitoring, quality control or audit of research involving human participants (persons appointed by the sponsor for quality control and audit; inspectors of the French regulatory authorities; representatives of the judicial authority), in accordance with applicable legal and regulatory provisions.

Persons with access to the processed data of persons taking part in the research, in accordance with applicable legal and regulatory provisions, within the limits of their authorisations with regard to their function and under conditions compliant with the regulations, take all necessary precautions to ensure the confidentiality of information relating to persons taking part in the research, particularly with regard to their identity and the results obtained. Persons accessing the data are bound by professional secrecy.

They may belong to the data controller, to the centres participating in the research or to structures acting on behalf of the sponsor (for example, investigators, persons responsible for quality control, clinical research assistants, auditors and any other person called upon to collaborate).

The sponsor will ensure that each person taking part in the research has agreed to access to the individual data concerning him or her that are strictly necessary for the quality control of the research.

## 13.4 Source documents

Source documents, defined as any original document or object that proves the existence or accuracy of a data item or fact recorded during the research, will be kept until two years after the last publication of the research results or, in the absence of publication, until the signature of the final research report. They will then be archived on paper or electronic media for a period of 15 years in accordance with applicable regulations, by the investigator or by the hospital in the case of a hospital medical record.

The source file will include the date and name of the physician who provided the information and any observations, the date on which the participant was informed, the participant’s code in the research and the medical data necessary for his or her follow-up, as well as the dates of visits and samples.

The following documents will be considered source documents:

- The medical record;
- The medical record adapted for the research, where applicable, including the data collected by the investigator and all traceability documents;
- Medical examination result sheets.

## 13.5 Data processing and retention of documents and data

The sole purpose of the processing of personal data of persons taking part in this research is the conduct of the research. This processing includes the management of data relating to persons taking part in the research, to allow the collection, entry, validity and consistency checking, and statistical analysis of the data collected during the research.

### 13.5.1 Description of the data collected

Only data that are strictly necessary and relevant to meet the objectives of this research will be collected. The data collected in this research fall into the following categories:

- **Identification:** in the form of an alphanumeric code assigned to each patient;
- **Epidemiological data:** age, sex, weight, height, body mass index, mRS before OHCA, Charlson comorbidity index (detailed in **addendum no. 5**) and Clinical Frailty Scale score;
- **OHCA history:** date, time and place of occurrence, presence or absence of a witness, bystander chest compressions or not, type of initial rhythm (shockable or non-shockable), external electrical shock and number of shocks, administration of adrenaline, noradrenaline as bolus or continuous infusion, fluid resuscitation, duration of “no-flow” and “low-flow”, time between OHCA onset and arrival of first responders, date and time of ROSC, date and time of ICU arrival, SBP/DBP/MAP/HR before inclusion, Glasgow score after ROSC;
- **Aetiological investigations:** aetiology finally retained for the cause of OHCA;
- **Parameters at inclusion (H0):** SBP, DBP, MAP, HR, temperature, Glasgow score, dose of catecholamines in progress (noradrenaline and/or adrenaline);
- **Parameters at ICU admission:** SBP, DBP, MAP, HR, temperature, Glasgow score, laboratory tests (*platelets, creatinine, total bilirubin, leucocytes, prothrombin time, urea, CRP, troponin, procalcitonin*), arterial blood gases (pH, PaCO_2_, PaO_2_, bicarbonate, arterial lactate, PaO_2_/FiO_2_), dose of catecholamines in progress; aetiology of the cardiac arrest;
- **Parameters at each RIC session or “sham session” (H0, H12 and H24):** thigh used, place of the session, date and time of start and end of session, reason for any interruption of the session, HR and MAP at the start and end of the session, dose of catecholamines at the start and end of the session, fluid resuscitation, occurrence of arterial hypotension during the session (MAP < 65 mmHg), occurrence of a complication during the session;
- **At the 6th hour (H6) after OHCA:** vital status, SBP, DBP, MAP, HR, temperature, Glasgow score, blood NfL, NSE and S100B measurements, dose of catecholamines in progress, fluid resuscitation, urine output;
- **At the 12th hour (H12) after OHCA:** vital status, SBP, DBP, MAP, HR, temperature, Glasgow score, dose of catecholamines in progress, fluid resuscitation, urine output;
- **At the 24th hour (H24) after OHCA:** vital status, SBP, DBP, MAP, HR, temperature, Glasgow score, calculation of the SAPS II score (detailed in **addendum no. 6**), NfL, NSE and S100B measurements, arterial blood gases (pH, PaCO_2_, PaO_2_/FiO_2_, bicarbonate, lactate), laboratory tests (platelets, creatinine, total bilirubin), dose of catecholamines in progress, SOFA, fluid resuscitation, urine output;
- **At the 48th hour (H48) after OHCA:** vital status, SBP, DBP, MAP, HR, temperature, Glasgow score, NfL, NSE and S100B measurements, arterial blood gases (pH, PaCO_2_, PaO_2_/FiO_2_, bicarbonate, lactate), laboratory tests (platelets, creatinine, total bilirubin), dose of catecholamines in progress, SOFA, fluid resuscitation, urine output;
- **At the 72nd hour (H72) after OHCA:** vital status, SBP, DBP, MAP, HR, temperature, Glasgow score, NfL, NSE and S100B measurements, arterial blood gases (pH, PaCO_2_, PaO_2_/FiO_2_, bicarbonate, lactate), laboratory tests (platelets, creatinine, total bilirubin), dose of catecholamines in progress, SOFA, fluid resuscitation, urine output;
- **Other therapies:** targeted temperature and device used to achieve it, use of ECMO, use of renal replacement therapy (RRT), coagulopathy support (transfusion of platelets or any coagulation factor);
- **Course during the ICU stay:** start and end dates of mechanical ventilation, catecholamines, RRT and ECMO;
- **Collection of post-OHCA complications** up to H72 after inclusion (unexpected cardiac arrest in the ICU, iatrogenic event, limb ischaemia, bowel obstruction, mesenteric ischaemia, thromboembolic complication, other);
- **In the event of death, date and mode of death:** recurrent unexpected cardiac arrest, refractory shock, respiratory failure, withdrawal of life-sustaining therapy for neurological failure, withdrawal of life-sustaining therapy for multi-organ failure, iatrogenic event, other;
- **At ICU discharge:** vital status and mRS assessed by the investigating physician, date of any decision to limit life-sustaining therapy;
- **At day 90 (D90) after inclusion:** vital status and mRS assessed by the investigating physician, location (still in the ICU, discharged from the ICU but still hospitalised, discharged from the ICU and currently in a rehabilitation facility, returned home), date of hospital discharge.

### 13.5.2 Case report form (eCRF)

All information required by the protocol must be recorded in the case report forms. Data must be collected as they are obtained and recorded explicitly in these forms.

This case report form will be implemented at the centre using an Internet-based data collection system (eCRF). Completion of the case report form via the Internet by the investigator allows the CRA to view the data rapidly and remotely.

The investigator is responsible for the accuracy, quality and relevance of all data entered. In addition, the data are checked immediately upon entry by means of consistency checks. The investigator must therefore validate any change of value in the eCRF. These changes are subject to an audit trail. A justification may be entered as a comment. The eCRF will be electronically signed by the investigator. An export of the eCRF will be performed and will constitute the research database. An electronic (CD-ROM) or paper copy will be provided by the sponsor to the investigator at the end of the research for archiving by the investigator. The sponsor’s internal procedures will be followed.

### 13.5.3 Data entry into the research database

Data will be entered electronically via the eCRF by members of the clinical centre authorised by the investigator. After training, these persons will receive access codes to the eCRF allowing data entry. An export of the eCRF will be performed and will constitute the research database.

### 13.5.4 Data processing, verification and validation procedures

After integration of any justified corrections to all data, the corresponding database will be frozen before being used for statistical analysis.

### 13.5.5 Data analysis

The person responsible for the analysis of the database is the Clinical Research and Innovation Department (DRCI) of CHITS.

### 13.5.6 Data retention

The database used for the statistical analysis is archived by the person responsible for the analysis and by the sponsor.

An electronic copy will be provided by the sponsor at the end of the research to each investigating centre, for the data concerning that centre, for archiving by the investigator.

The sponsor’s internal procedures will be followed.

## 13.6 Archiving

The data collected in the research will be kept until two years after the last publication of the research results or, in the absence of publication, until the signature of the final research report. They will then be archived on paper or electronic media for a period of 15 years in accordance with applicable regulations. Investigators will ensure that source files, and in particular medical records, are available throughout this archiving period.

This indexed archive will comply with GCP and will include (non-exhaustive list):

- The signed consent forms, where applicable, of all persons at the centre who took part in the research, for the investigator;
- The “research” files for the investigator and the sponsor, including:
  - Successive versions of the protocol (identified by version number and date) and its appendices;
  - Copies of authorisation letters/opinions from regulatory bodies;
  - Correspondence;
  - The completed and validated CRF of each included participant;
  - The identification list of selected patients and the list of selected patients;
  - The summary of the final research report.

The database used for the statistical analysis must also be archived by the person responsible for the analysis (electronic media).

These documents serve to demonstrate that all parties involved in the research comply with the rules of Good Clinical Practice and with applicable legal and regulatory texts.

# 14 ETHICAL AND REGULATORY CONSIDERATIONS

The Centre Hospitalier Intercommunal de Toulon – La Seyne sur Mer is the sponsor of this research.

This research will be conducted in compliance with applicable regulations, in particular the provisions relating to research involving human participants set out in Articles L1121-1 et seq. of the French Public Health Code, the Bioethics laws, the French Data Protection Act (Loi Informatique et Libertés), the General Data Protection Regulation (GDPR), GCP and this protocol.

| **Tasks** | **Responsible structure** | **Details** |
| --- | --- | --- |
| **Inclusions, collection of consent** | SAMU 83 – CHITS  Polyvalent Intensive Care Unit – CHITS | Physicians in charge of the patient |
| **Dispatch of biological samples** | Medical Biology Laboratory – CHITS |  |
| **Analysis of biological samples** | Medical Biology Laboratory – CHITS  Medical Biology Laboratory – CHU de Clermont-Ferrand | Routine care laboratory tests and blood NSE measurement.  Blood NfL and S100B measurements |

## 14.1 Ethics committees

### 14.1.1 Comité de Protection des Personnes (CPP)

Before the start of the research in France, for research involving human participants not relating to a health product referred to in Article L5311-1, the sponsor obtains the favourable opinion of a Comité de Protection des Personnes (CPP) designated at random under the conditions set out in Article L1123-14 of the French Public Health Code and will provide it with all the necessary information for this purpose.

The CPP Est IV issued a favourable opinion for this research on 8 April 2024.

Any substantial modification of the submitted documents must be the subject of a written amendment, which will be submitted to the research steering committee and to the sponsor for approval. Before its implementation, the sponsor must obtain the favourable opinion of the CPP.

## 14.2 French National Agency for the Safety of Medicines and Health Products (ANSM)

The summary of the research will be sent to the ANSM for information.

## 14.3 French Data Protection Authority (CNIL)

The computer file used for this research is implemented in accordance with French (amended French Data Protection Act) and European (GDPR) regulations.

This research does not fall within the scope of the CNIL “Reference Methodology” (MR-001), because patients are to be included in an emergency situation without their consent being obtained at inclusion. Before implementing the processing of the data necessary to conduct the research, the sponsor must obtain authorisation from the CNIL.

## 14.4 Data ownership

The sponsor owns the data, and no use or transfer to a third party may be made without its prior agreement.

## 14.5 Protocol amendments

The coordinating investigator informs the sponsor of any proposed modification of the protocol. Any substantial modification will be submitted by the sponsor to the competent authorities and ethics committees for opinion and/or authorisation. A substantial modification is a modification likely, in one way or another, to change the guarantees provided to persons taking part in the research (change to an inclusion criterion, extension of an enrolment period, participation of new centres, etc.). Any extension of the research (major change in the treatment regimen or in the populations included, extension of treatments and/or therapeutic procedures not initially planned in the protocol) must be considered as new research.

The information sheets and consent forms may be revised if necessary, particularly in the event of a substantial modification of the research or the occurrence of adverse effects.

All protocol amendments must be brought to the attention of all investigators participating in the research. Investigators undertake to comply with their content.

# 15 FUNDING AND INSURANCE

## 15.1 Source of funding

The research is funded in the amount of €15,000 through the Extra-University grant of the Société de Réanimation de Langue Française (SRLF); in addition, co-funding is provided by CHITS.

The source of funding will be mentioned in the acknowledgements of all publications.

## 15.2 Insurance

The sponsor has taken out insurance (policy no. 132.515) with Relyens for the entire duration of the research, covering its own civil liability as well as that of any physician or staff member involved in conducting the research.

# 16 PUBLICATION OF RESULTS AND FINAL REPORT

## 16.1 Final report

The final report of the research involving human participants referred to in Article R1123-60 of the CSP will be written jointly by the sponsor, the coordinating investigator and the biostatistician of this research. This report will be submitted to each investigator for opinion. Once consensus has been reached, the final version must be endorsed by the signature of each investigator and sent to the sponsor as soon as possible after the effective end of the research, and at the latest within 9 months of the end of the research (planned or early termination).

A summary of the report, written according to the reference outline of the competent authority, must be sent to the competent authority within one year after the end of the research, corresponding to the end of participation of the last person taking part in the research. This time limit is reduced to 90 days in the event of early termination of the research.

## 16.2 Communication of research results to participants

Participants who have expressed the wish will be informed of the overall results by the investigating centre by means of a letter written and submitted to the CPP for information, based on the summary of the final research report.

## 16.3 Scientific communication

Any publication, whether complete or partial, of the results of this research (manuscripts, abstracts, press articles, oral presentations or posters, etc.) by the investigators or their representatives requires the approval of the coordinating investigator and of the research steering committee. Unless specifically agreed by the coordinating investigator and the steering committee, no part of the research may be presented as a poster or oral presentation before acceptance of the corresponding manuscript in a peer-reviewed journal.

It is planned to publish the results of the study in an original article in an appropriate medical journal and to present them at international congresses. The choice of journal for publication will be made by the coordinating investigator and the steering committee in agreement with the co-authors.

In accordance with the 2017 update of the recommendations of the International Committee of Medical Journal Editors (http://www.icmje.org/), authorship will be based on the following four criteria:

- Substantial contributions to the conception or design of the work, or the acquisition, analysis or interpretation of data; AND
- Drafting the work or revising it critically for important intellectual content; AND
- Final approval of the version to be published; AND
- Agreement to be accountable for all aspects of the work in ensuring that questions related to the accuracy or integrity of any part of the work are appropriately investigated and resolved.

The coordinating investigator and the research steering committee will decide on the names and order of authors on the publication. The first author and corresponding author will be Dr Jonathan Chelly.

The authors of any publication related to this research must:

- Name CHITS as sponsor;
- For authors belonging to CHITS, include CHITS in their affiliations;
- Cite the AfterROSC research group as providing scientific and methodological expertise in the “Acknowledgements” section of the article;
- Cite the SRLF as a funder in the “Acknowledgements” section of the article;
- Cite any other organisation funding the study in the “Acknowledgements” section of the article.

Accordingly, the investigator must send a copy of his or her communications and/or publications to the CHITS Clinical Research and Innovation Department – Sponsorship Unit.

The authors will indicate the source of funding of the study by adding the following statement: “This research is funded by ….”.

This research will be registered on the http://clinicaltrials.gov/ platform.

## 16.4 References

1.	Lemiale V, Dumas F, Mongardon N, Giovanetti O, Charpentier J, Chiche JD, et al. Intensive care unit mortality after cardiac arrest: the relative contribution of shock and brain injury in a large cohort. Intensive Care Med. 2013 Nov;39(11):1972–80.

2.	Negovsky VA. Postresuscitation disease. Crit Care Med. 1988;16(10):942–6.

3.	Adrie C, Laurent I, Monchi M, Cariou A, Dhainaou JF, Spaulding C. Postresuscitation disease after cardiac arrest: a sepsis-like syndrome? Curr Opin Crit Care. 2004 Jun;10(3):208–12.

4.	Nolan JP, Sandroni C, Böttiger BW, Cariou A, Cronberg T, Friberg H, et al. European Resuscitation Council and European Society of Intensive Care Medicine guidelines 2021: post-resuscitation care. Intensive Care Med. 2021 Apr;47(4):369–421.

5.	Ho AFW, Chong J, Ong MEH, Hausenloy DJ. Remote Ischemic Conditioning in Emergency Medicine—Clinical Frontiers and Research Opportunities. Shock. 2020 Mar;53(3):269–76.

6.	Cour M, Gomez L, Mewton N, Ovize M, Argaud L. Postconditioning: From the Bench to Bedside. J Cardiovasc Pharmacol Ther. 2011 Jun;16(2):117–30.

7.	Kloner RA, Shi J, Dai W, Carreno J, Zhao L. Remote Ischemic Conditioning in Acute Myocardial Infarction and Shock States. J Cardiovasc Pharmacol Ther. 2020 Mar;25(2):103–9.

8.	Thielmann M, Kottenberg E, Kleinbongard P, Wendt D, Gedik N, Pasa S, et al. Cardioprotective and prognostic effects of remote ischaemic preconditioning in patients undergoing coronary artery bypass surgery: a single-centre randomised, double-blind, controlled trial. Lancet. 2013 Aug 17;382(9892):597–604.

9.	Gaspar A, Lourenço AP, Pereira MÁ, Azevedo P, Roncon-Albuquerque R, Marques J, et al. Randomized controlled trial of remote ischaemic conditioning in ST-elevation myocardial infarction as adjuvant to primary angioplasty (RIC-STEMI). Basic Res Cardiol. 2018 May;113(3):14.

10.	Sloth AD, Schmidt MR, Munk K, Kharbanda RK, Redington AN, Schmidt M, et al. Improved long-term clinical outcomes in patients with ST-elevation myocardial infarction undergoing remote ischaemic conditioning as an adjunct to primary percutaneous coronary intervention. Eur Heart J. 2014 Jan 14;35(3):168–75.

11.	England TJ, Hedstrom A, O’Sullivan SE, Woodhouse L, Jackson B, Sprigg N, et al. Remote Ischemic Conditioning After Stroke Trial 2: A Phase IIb Randomized Controlled Trial in Hyperacute Stroke. J Am Heart Assoc. 2019 Dec 3;8(23):e013572.

12.	Lu M, Wang Y, Yin X, Li Y, Li H. Cerebral protection by remote ischemic post-conditioning in patients with ischemic stroke: A systematic review and meta-analysis of randomized controlled trials. Front Neurol. 2022 Sep 21;13:905400.

13.	Chen HS, Cui Y, Li XQ, Wang XH, Ma YT, Zhao Y, et al. Effect of Remote Ischemic Conditioning vs Usual Care on Neurologic Function in Patients With Acute Moderate Ischemic Stroke: The RICAMIS Randomized Clinical Trial. JAMA. 2022 Aug 16;328(7):627.

14.	Joseph B, Pandit V, Zangbar B, Kulvatunyou N, Khalil M, Tang A, et al. Secondary brain injury in trauma patients: The effects of remote ischemic conditioning. J Trauma Acute Care Surg. 2015 Apr;78(4):698–705.

15.	Cour M, Klouche K, Souweine B, Quenot JP, Schwebel C, Perinel S, et al. Remote ischemic conditioning in septic shock: the RECO-Sepsis randomized clinical trial. Intensive Care Med. 2022 Nov;48(11):1563–72.

16.	Jensen HA, Loukogeorgakis S, Yannopoulos F, Rimpiläinen E, Petzold A, Tuominen H, et al. Remote Ischemic Preconditioning Protects the Brain Against Injury After Hypothermic Circulatory Arrest. Circulation. 2011 Feb 22;123(7):714–21.

17.	Albrecht M, Meybohm P, Broch O, Zitta K, Hein M, Gräsner JT, et al. Evaluation of remote ischaemic post-conditioning in a pig model of cardiac arrest: A pilot study. Resuscitation. 2015 Aug;93:89–95.

18.	Bartlett E, Morse SC, Morse D, Goodson L, Adams KN, Nichol G. Randomized Feasibility Trial of Remote Ischemic Conditioning to Enhance Resuscitation (RICE). Resuscitation. 2023 Oct 13;110003.

19.	Benjamin EJ, Blaha MJ, Chiuve SE, Cushman M, Das SR, Deo R, et al. Heart Disease and Stroke Statistics—2017 Update: A Report From the American Heart Association. Circulation [Internet]. 2017 Mar 7 [cited 2022 Jul 11];135(10). Available from: https://www.ahajournals.org/doi/10.1161/CIR.0000000000000485

20.	Atwood C, Eisenberg MS, Herlitz J, Rea TD. Incidence of EMS-treated out-of-hospital cardiac arrest in Europe. Resuscitation. 2005 Oct;67(1):75–80.

21.	Bougouin W, Lamhaut L, Marijon E, Jost D, Dumas F, Deye N, et al. Characteristics and prognosis of sudden cardiac death in Greater Paris: Population-based approach from the Paris Sudden Death Expertise Center (Paris—SDEC). Intensive Care Med. 2014;40(6):846–54.

22.	Daya MR, Schmicker RH, Zive DM, Rea TD, Nichol G, Buick JE, et al. Out-of-hospital cardiac arrest survival improving over time: Results from the Resuscitation Outcomes Consortium (ROC). Resuscitation. 2015 Jun;91:108–15.

23.	Wong MKY, Morrison LJ, Qiu F, Austin PC, Cheskes S, Dorian P, et al. Trends in Short- and Long-Term Survival Among Out-of-Hospital Cardiac Arrest Patients Alive at Hospital Arrival. Circulation. 2014 130(21):1883–90.

24.	Humaloja J, Ashton NJ, Skrifvars MB. Brain Injury Biomarkers for Predicting Outcome After Cardiac Arrest. Crit Care. 2022 Mar 22;26(1):81.

25.	Adler C, Onur OA, Braumann S, Gramespacher H, Bittner S, Falk S, et al. Absolute serum neurofilament light chain levels and its early kinetics predict brain injury after out-of-hospital cardiac arrest. J Neurol. 2022 Mar;269(3):1530–7.

26.	Moseby-Knappe M, Mattsson-Carlgren N, Stammet P, Backman S, Blennow K, Dankiewicz J, et al. Serum markers of brain injury can predict good neurological outcome after out-of-hospital cardiac arrest. Intensive Care Med. 2021 Sep;47(9):984–94.

27.	Moseby-Knappe M, Mattsson N, Nielsen N, Zetterberg H, Blennow K, Dankiewicz J, et al. Serum Neurofilament Light Chain for Prognosis of Outcome After Cardiac Arrest. JAMA Neurol. 2019 Jan 1;76(1):64.

# 17 ADDENDA

1.	**Simplified overview of the cellular mechanisms of action of ischaemic post-conditioning**

2.	**Sequential Organ Failure Assessment (SOFA) score**

3.	**Modified Rankin Scale (mRS)**

4.	**Glasgow Coma Scale**

5.	**Charlson comorbidity index**

6.	**Simplified Acute Physiology Score II (SAPS II)**

7.	**Information sheet and consent form – “relative”**

8.	**Information sheet and continuation consent form – “patient”**

9.	**Emergency inclusion form**

**Notes:**

- Each addendum and the register of addendum versions are attached separately from the protocol.
- Each addendum may be modified (change of addendum version) without changing the protocol version.

## Addendum no. 1: Simplified overview of the cellular mechanisms of action of ischaemic post-conditioning

**Simplified overview of the cellular mechanisms of action of ischaemic post-conditioning** (Post-C). Post-C inhibits glycogen synthase kinase-3β (GSK-3β) *via* the activation of membrane receptors by autacoids (adenosine, bradykinin, etc.). Post-C may also protect the cell *via* the maintenance of intracellular acidosis during the reperfusion phase and by modulating the formation of cytotoxic reactive oxygen species. All these active or passive mechanisms lead to inhibition of the mitochondrial permeability transition pore (mPTP), which underlies the cellular protective effect (adapted from *Cour et al. Postconditioning: from the bench to bedside. J Cardiovasc Pharmacol Ther 2011;16:117–30*).

## Addendum no. 2: Sequential Organ Failure Assessment (SOFA) score

| **Organ parameters** | **0 points** | **1 point** | **2 points** | **3 points** | **4 points** |
| --- | --- | --- | --- | --- | --- |
| **PaO_2_/FiO_2_ (mmHg)** | > 400 | 301–400 | 201–300 | 101–200 with ventilatory support | ≤ 100 with ventilatory support |
| **Platelets (×10^3^/mm^3^)** | > 150 | 101–150 | 51–100 | 21–50 | ≤ 20 |
| **Bilirubin (µmol/L)** | < 20 | 20–32 | 33–101 | 102–204 | > 204 |
| **Hypotension** | MAP ≥ 70 mmHg | MAP < 70 mmHg | Dopamine ≤ 5 or dobutamine (any dose) | Dopamine > 5 or adrenaline/noradrenaline ≤ 0.1 | Dopamine > 15 or adrenaline/noradrenaline > 0.1 |
| **Glasgow Coma Scale** | 15 | 13–14 | 10–12 | 6–9 | < 6 |
| **Creatinine (µmol/L) or urine output** | < 110 | 110–170 | 171–299 | 300–440 or urine output < 500 mL/day | > 440 or urine output < 200 mL/day |
| MAP: mean arterial pressure. Catecholamine doses in µg/kg/min. | | | | | |

## Addendum no. 3: Modified Rankin Scale (mRS)

| **Score** | **Description** |
| --- | --- |
| **0** | No symptoms |
| **1** | Minimal symptoms: not interfering with usual daily activities |
| **2** | Slight disability: restriction of some daily activities, but patient independent |
| **3** | Moderate disability: requiring some help, but able to walk without assistance |
| **4** | Moderately severe disability: unable to walk without assistance, marked restriction of independence but without need for constant assistance |
| **5** | Severe disability: bedridden, incontinent and requiring constant nursing care |
| **6** | Death |

## Addendum no. 4: Glasgow Coma Scale

| **Eye opening** | **Verbal response** | **Motor response** |
| --- | --- | --- |
| Spontaneous = **4** | Oriented = **5** | Obeys commands = **6** |
| To voice = **3** | Confused = **4** | Localises to pain = **5** |
| To pain = **2** | Inappropriate words = **3** | Withdraws from pain = **4** |
| None = **1** | Incomprehensible sounds = **2** | Abnormal flexion = **3** |
|  | None = **1** | Extension = **2** |
|  |  | None = **1** |

## Addendum no. 5: Charlson comorbidity index

| **Item** | **Weight** |
| --- | --- |
| Myocardial infarction | 1 point |
| Congestive heart failure | 1 point |
| Peripheral vascular disease | 1 point |
| Cerebrovascular disease (except hemiplegia) | 1 point |
| Dementia | 1 point |
| Chronic pulmonary disease | 1 point |
| Connective tissue disease | 1 point |
| Peptic ulcer disease | 1 point |
| Diabetes without complications | 1 point |
| Mild liver disease | 1 point |
| Hemiplegia | 2 points |
| Moderate to severe renal disease | 2 points |
| Diabetes with end-organ damage | 2 points |
| Cancer | 2 points |
| Leukaemia, lymphoma, multiple myeloma | 2 points |
| Moderate to severe liver disease | 3 points |
| Metastatic solid tumour | 6 points |
| AIDS | 6 points |

## Addendum no. 6: Simplified Acute Physiology Score II (SAPS II)

| **Variable** | **Categories (points)** |
| --- | --- |
| Age (years) | < 40 (0); 40–59 (7); 60–69 (12); 70–74 (15); 75–79 (16); ≥ 80 (18) |
| Heart rate (bpm) | < 40 (11); 40–69 (2); 70–119 (0); 120–159 (4); ≥ 160 (7) |
| Systolic blood pressure (mmHg) | < 70 (13); 70–99 (5); 100–199 (0); ≥ 200 (2) |
| Temperature (°C) | < 39 (0); ≥ 39 (3) |
| PaO_2_/FiO_2_ (mmHg), if mechanical ventilation or CPAP | < 100 (11); 100–199 (9); ≥ 200 (6) |
| Urine output (L/day) | < 0.5 (11); 0.5–0.99 (4); ≥ 1 (0) |
| Blood urea (mmol/L) | < 10 (0); 10–29.9 (6); ≥ 30 (10) |
| White blood cell count (×10^3^/mm^3^) | < 1 (12); 1–19.9 (0); ≥ 20 (3) |
| Serum potassium (mmol/L) | < 3 (3); 3–4.9 (0); ≥ 5 (3) |
| Serum sodium (mmol/L) | < 125 (5); 125–144 (0); ≥ 145 (1) |
| Serum bicarbonate (mmol/L) | < 15 (6); 15–19 (3); ≥ 20 (0) |
| Bilirubin (µmol/L) | < 68.4 (0); 68.4–102.5 (4); > 102.5 (9) |
| Glasgow Coma Scale | < 6 (26); 6–8 (13); 9–10 (7); 11–13 (5); 14–15 (0) |
| Chronic disease | Metastatic cancer (9); haematological malignancy (10); AIDS (17) |
| Type of admission | Scheduled surgical (0); medical (6); unscheduled surgical (8) |

Points for each category are shown in brackets.
